# Transposons played a major role in the diversification between the closely related almond and peach genomes: results from the almond genome sequence

**DOI:** 10.1111/tpj.14538

**Published:** 2019-10-22

**Authors:** Tyler Alioto, Konstantinos G. Alexiou, Amélie Bardil, Fabio Barteri, Raúl Castanera, Fernando Cruz, Amit Dhingra, Henri Duval, Ángel Fernández i Martí, Leonor Frias, Beatriz Galán, José L. García, Werner Howad, Jèssica Gómez‐Garrido, Marta Gut, Irene Julca, Jordi Morata, Pere Puigdomènech, Paolo Ribeca, María J. Rubio Cabetas, Anna Vlasova, Michelle Wirthensohn, Jordi Garcia‐Mas, Toni Gabaldón, Josep M. Casacuberta, Pere Arús

**Affiliations:** ^1^ CNAG‐CRG, Centre for Genomic Regulation (CRG) Barcelona Institute of Science and Technology (BIST) Baldiri i Reixac 4 08028 Barcelona Spain; ^2^ Universitat Pompeu Fabra (UPF) 08005 Barcelona Spain; ^3^ IRTA, Campus UAB Edifici CRAG Cerdanyola del Vallès (Bellaterra) 08193 Barcelona Spain; ^4^ Centre for Research in Agricultural Genomics (CRAG) CSIC‐IRTA‐UAB‐UB, Campus UAB Edifici CRAG Cerdanyola del Vallès (Bellaterra) 08193 Barcelona Spain; ^5^ Department of Horticulture Washington State University 99164-6414 Pullman WA USA; ^6^ INRA, UR1052 Unité de Génétique et Amélioration des Fruits et Légumes (GAFL) Domaine St. Maurice CS 60094 84143 Montfavet Cedex France; ^7^ Department of Environmental Science Policy and Management University of California Berkeley 94720 CA USA; ^8^ Innovative Genomics Institute (IGI) 94720 Berkeley CA USA; ^9^ Department of Environmental Biology Center for Biological Research (CIB‐CSIC) Spanish National Research Council (CSIC) Ramiro de Maeztu 9 28040 Madrid Spain; ^10^ Bioinformatics and Genomics Programme Centre for Genomic Regulation (CRG) Dr Aiguader, 88 08003 Barcelona Spain; ^11^ The Pirbright Institute Woking Surrey GU24 0NF UK; ^12^ Centro de Investigación y Tecnología Agroalimentaria de Aragón (CITA) Unidad de Hortofruticultura Gobierno de Aragón, Avda. Montañana 930 50059 Zaragoza Spain; ^13^ Instituto Agroalimentario de Aragón – IA2 (CITA‐Universidad de Zaragoza) Calle Miguel Servet 177 50013 Zaragoza Spain; ^14^ University of Adelaide Waite Research Institute School of Agriculture, Food and Wine PMB 1 Glen Osmond SA 5064 Australia; ^15^ Institució Catalana de Recerca i Estudis Avançats (ICREA) Pg Lluís Companys 23 08010 Barcelona Spain

**Keywords:** *Prunus dulcis*, *Prunus persica*, genome sequence, variability, divergence, indels, transposable elements, crop evolution, seed bitterness

## Abstract

We sequenced the genome of the highly heterozygous almond *Prunus dulcis* cv. Texas combining short‐ and long‐read sequencing. We obtained a genome assembly totaling 227.6 Mb of the estimated almond genome size of 238 Mb, of which 91% is anchored to eight pseudomolecules corresponding to its haploid chromosome complement, and annotated 27 969 protein‐coding genes and 6747 non‐coding transcripts. By phylogenomic comparison with the genomes of 16 additional close and distant species we estimated that almond and peach (*Prunus persica*) diverged around 5.88 million years ago. These two genomes are highly syntenic and show a high degree of sequence conservation (20 nucleotide substitutions per kb). However, they also exhibit a high number of presence/absence variants, many attributable to the movement of transposable elements (TEs). Transposable elements have generated an important number of presence/absence variants between almond and peach, and we show that the recent history of TE movement seems markedly different between them. Transposable elements may also be at the origin of important phenotypic differences between both species, and in particular for the sweet kernel phenotype, a key agronomic and domestication character for almond. Here we show that in sweet almond cultivars, highly methylated TE insertions surround a gene involved in the biosynthesis of amygdalin, whose reduced expression has been correlated with the sweet almond phenotype. Altogether, our results suggest a key role of TEs in the recent history and diversification of almond and its close relative peach.

## Significance Statement

Almond and peach are closely related and cross‐compatible species of the genus *Prunus* that separated about 6 million years ago. Here we show that the almond genome, with 227.6 Mbp and 27 696 protein‐coding genes, is highly similar to that of peach. However, transposable elements have been recently active and have generated mutations that may be responsible for some key phenotypic differences between peach and almond, such as the sweet versus bitter almond taste.

## Introduction

Almond, *Prunus dulcis* (Miller) D.A. Webb (syn. *Prunus amygdalus* Batsch), is a rosaceous tree species cultivated for its seeds; it has a diploid (2*n* = 2*x* = 16) and compact genome (about 300 Mbp) (Baird *et al.*, 1994). The genus *Prunus* comprises a group of approximately 200 species, some of which, such as the stone fruits (peach, apricot, cherry and plum) and almond, have high economic value (Aranzana *et al.*, 2019). The high level of genomic resemblance and synteny among the species of this genus (Dirlewanger *et al.*, 2004) enables production of hybrids that are sometimes fertile.

Humans used almond as a food long before the advent of agriculture, and the oldest remains have been found in Israel, dating from 19 000 years ago (Kislev *et al.*, 1992), although its domestication probably occurred 14 000 years later (Spiegel‐Roy, 1976). The origin of the almond tree is not well established; its closest wild relatives live in central and western Asia, stretching from the Himalayas to the eastern Mediterranean Basin (Yazbek and Al‐Zein, 2009). Based on the distribution of the cultivated species, two alternative hypotheses place the domestication site of almond in the Levant (Browicz and Zohary, 1996) or in central Asia (Ladizinsky, 1999). Diamond (1997) proposed almond as an example of simple domestication, where a dominant mutation at a single gene conferring a sweet taste to the otherwise bitter and toxic kernel would result in an edible and cultivable crop. This gene, sweet kernel *Sk*/*sk*, was initially described by Heppner (1923) and later mapped to the central region of chromosome 5 (Sánchez‐Pérez *et al.*, 2007); it has recently been proposed to correspond to a basic helix–loop–helix (bHLH) transcription factor (Sánchez‐Pérez *et al.*, 2019). The closest relatives of almond are within the subgenus *Amygdalus*, encompassing peach [*Prunus persica* (L.) Batsch] and a group of 25 wild species (Yazbek and Al‐Zein, 2009). Peach and almond hybrids are fertile. In fact, peach was proposed by Darwin (1968) as a possible direct derivative of almond with a fleshy, non‐dehiscent and juicy mesocarp. However, molecular phylogenetics has identified a clear separation between peach and almond consistent with their geographical origin and distribution: peach and its closest relatives are native to China and eastern Asia, whereas almond and its wild relatives are native to central and western Asia (Delplancke *et al.*, 2016).

The genome sequences of some *Prunus* species are available, including the high‐quality genome of peach (Verde *et al.*, 2017) and those of sweet cherry (*Prunus avium* L.) (Shirasawa *et al.*, 2017), mume (*Prunus mume* L.), a relative of apricot (Zhang *et al.*, 2017), and *Prunus yedoensis*, a wild cherry tree (Baek *et al.*, 2018), the latter two used for ornamental purposes. The genome sequence of almond cv. Lauranne has been very recently added (Sánchez‐Pérez *et al.*, 2019). In this paper, we present the whole genome sequence of almond cv. Texas, a self‐incompatible and highly heterozygous genotype that was obtained in the USA from materials imported from western Europe. Texas (also called Texas Prolific and Mission) was bred at Houston, Texas, USA, as a seedling of French cultivar Languedoc (Wickson, 2001), and became one of the leading cultivars in California in the twentieth century along with ‘Nonpareil’ (Kester *et al.*, 2015). Texas was also one of the parents, the other was peach cv. Earlygold, of the interspecific progeny used for the construction of the reference linkage map of *Prunus* (Joobeur *et al.*, 1998). We also compared the Texas almond genome sequence with other sequenced genomes, including that of its close relative peach, and found that, in addition to other aspects of diversity between these genomes already reported (Yu *et al.*, 2001; Velasco *et al.*, 2014), transposable elements (TEs) played a key role in their recent diversification.

## Results

### Texas almond sequence assembly, annotation and comparison with the linkage map and the peach sequence

A total of 138.6 Gb of Illumina (>500× coverage) and 10.2 Gb (37×) of Oxford Nanopore Technologies (ONT; score ≥ 7.0 and a read N50 length of 7.3 kb) sequence were produced (Table S1 in the online Supporting Information). By analyzing *k*‐mer frequency, the lower bound for genome size was estimated to be 238 Mb (Figure S1). We collapsed the assembly into a haploid representation and anchored it to eight pseudomolecules, the number of the haploid almond chromosome complement. The final assembly, *P. dulcis* Texas v.2.0 (also known as pdulcis26) totals 227.6 Mb (91.5% of which is anchored to the eight pseudomolecules) and has a contig and scaffold N50s of 103.9 and 381.5 kb, respectively (Table 1). The completeness of the assembly as determined by BUSCO analysis is 96.4%: 95.4% complete (89.4% unique and 6.0% duplicated), 1.0% fragmented and 3.6% missing BUSCOs. *k*‐mer analysis confirmed the BUSCO results (Figure S1).

**Table 1:** Texas genome assembly and annotation statistics

Assembly length	227.6 Mb
Contig N50	103.9 kb
Scaffold N50	381.5 kb
Pseudomolecule N50	24.8 Mb
Per cent anchored to pseudomolecules	91.47%
BUSCO complete genes	95.4%
BUSCO fragmented genes	1.0%
BUSCO missing genes	3.6%
Genomic GC content	37.65%
Number of protein‐coding genes	27 969
Median gene length (bp)	2288
Number of transcripts	34 039
Number of unique protein products	32 559
Number of exons	184 149
Number of unique exons	148 374
Number of coding exons	140 538
Coding GC content	44.12%
Median intron length (bp)	171
Exons/transcript	5.41
Transcripts/gene	1.22
Multi‐exonic transcripts	81%
Gene density (genes Mb^–1^)	123

We annotated a total of 27 969 protein‐coding genes that produce 34 039 transcripts (1.22 transcripts per gene) and encode for 32 559 unique protein products (Table 1). We were able to assign some type of functional annotation to 92% of them. In addition, we annotated 6747 non‐coding transcripts, of which 3590 and 3153 are long and short non‐coding RNA genes, respectively. Most of the main assembly and annotation parameters of the Texas genome presented here were similar to those obtained by Sánchez‐Pérez *et al. *(2019) on cv. Lauranne (see Table S2).

Out of the 1833 single nucleotide polymorphisms (SNPs) that comprise the Texas × Earlygold (T×E) linkage map (Donoso *et al.*, 2015), 1609 (87.8%) mapped onto the almond assembly with single high‐quality hits (percentage identity ≥ 90% and marker coverage ≥ 90%) (Table S3), with 1597 (93.4%) mapping onto the anchored assembly. Of the anchored SNPs, 1578 aligned with the pseudomolecules and were syntenic and collinear to the eight chromosomes of peach. Only 19 SNPs had a different order on the assembly, two of which mapped to a pseudomolecule that was different from the linkage group of the T×E map and 10 on an unassigned scaffold (Table S3, Figure S2), which we attributed to contig reordering or minor misassemblies. Similarly, we observed high synteny and collinearity between the genome sequence of peach v.2.0 a1 (Verde *et al.*, 2002) and that of the Texas almond generated here (Figure S3). A comparison of physical versus genetic distances of the eight pseudomolecules is presented in Figure S4. Regions of low recombination rates usually coincide with pericentromeric regions and occurred at similar regions to those in the peach genome (Verde *et al.*, 2017).

### Phylogenomic analysis

To shed light on the evolutionary history of the genome of *P. dulcis* in the context of 16 other sequenced plant species (Table S4), we generated the phylomes of almond and peach, that is their complete collections of gene phylogenies (see Experimental Procedures). These phylomes were filtered to remove the gene trees containing proteins with domains associated with transposons. After filtering, a total of 18 475 and 20 812 gene trees were kept for almond and peach, respectively. These filtered phylomes were scanned to infer duplications and speciation events and derive orthology and paralogy relationships from individual gene trees (Gabaldón, 2008). These analyses produced a catalog of gene duplication events and phylogeny‐based homology relationships for genes in the 17 considered species, which were used in subsequent analyses.

**Figure 1:**
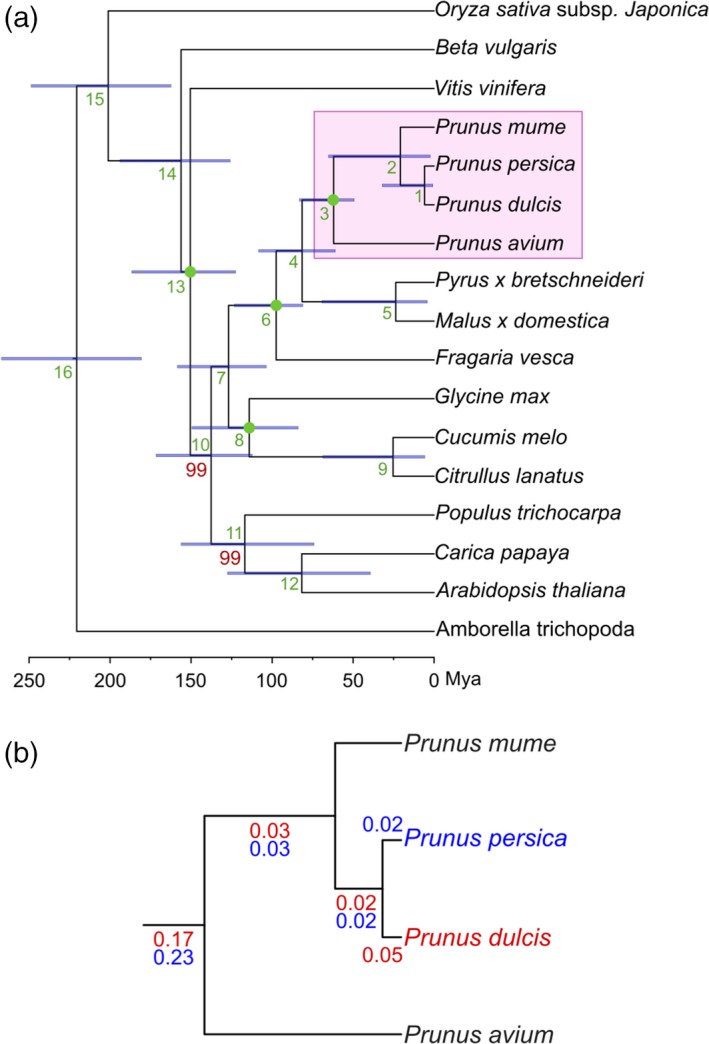
Species tree obtained from the concatenation of 262 widespread single‐gene families. (a) Full species tree. All *Prunus* species are highlighted in pink. All bootstrap values that are not maximal (bootstrap 100%) are indicated in red. Green numbers correspond to the nodes in Table S4. Bars at the nodes indicate the uncertainty around mean age estimates based on 95% credibility intervals. Scale at the bottom shows the divergence time in Mya (million years ago). Green dots represent selected calibration points. (b) Zoom‐in of the *Prunus* group. Numbers indicate the duplication ratio for each branch calculated with the phylome of almond (red) and peach (blue).

We concatenated the protein alignments of 262 genes that had single‐copy orthologs in all the 17 species considered to reconstruct a phylogeny of these species. The resulting highly supported topology (Figure 1a) was congruent with current views on plant phylogeny (Shaw and Small, 2004) and results in *P. dulcis* and *P. persica* forming a clade, to the exclusion of *P. mume* and *P. avium* (Badenes and Parfitt, 1995; Scholz *et al.*, 2013)*.* The same topology was obtained when all individual gene trees were combined into a single species phylogeny by using a gene tree parsimony approach. We estimated the divergence times on this topology using a Bayesian relaxed molecular clock approach. According to our results, *P. dulcis* diverged from *P. persica* approximately 5.88 million years ago (Mya), from *P. mume* 20.84 Mya and from *P. avium* 62.04 Mya (Figure 1a, Table S5).

We then calculated the duplication frequency (i.e. the average number of duplications per gene) for each node of the species tree, and observed a slightly high duplication frequency (about 0.20; Figure 1b) at the common ancestor of all *Prunus* species, which is supported by both almond and peach phylomes. A functional analysis of protein families duplicated at this branch shows enrichment of some molecular functions such as methyltransferase activity, ionotropic glutamate receptor activity, terpene synthase activity, oxidoreductase activity and transferase activity. In addition, some biological processes were enriched: response to auxin, metabolic process and oxidation–reduction process (Table S6).

Then we focused on duplications specific to almond or peach, including large expansions. A total of 1175 (4.4%) almond proteins and 831 (3.1%) peach proteins have an in‐paralog (a recent paralog resulting from a duplication that specifically occurred in the almond and peach lineage, respectively). These paralogs could be assigned to 542 almond‐specific gene expansions and 367 peach‐specific gene expansions. In both almond and peach most expansions [540 (99.6%) for almond; 363 (98.9%) for peach] have a moderate size (two to five in‐paralogs; Figure S5). Some almond expansions of size two encode putative members of the lignin biosynthesis pathway (Vanholme *et al.*, 2010) such as caffeic acid 3‐*O*‐methyltransferase (COMT; Prudul26A009858P1‐Prudul26A011895P1) and shikimate *O*‐hydroxycinnamoyl transferase (HCT; Prudul26A003924P1‐Prudul26A028947P1, Prudul26A000852P1‐Prudul26A022843P2). Interestingly, these genes have undergone parallel duplications in *P. persica*, *P. mume* and *P. avium* that occurred independently in the three species. In order to check whether the almond species‐specific duplications are tandem or dispersed duplications we assessed whether they were present in the same or different scaffolds, and if present in the same scaffold we counted the number of genes that are present between the resulting in‐paralogs. From the total number of species‐specific paralogous pairs or in‐paralogs (732), the majority (384, 52%) were located in the same scaffold, and 285 pairs (39%) were located in close proximity, with fewer than 10 intervening genes. A group of 87 (12%) paralogs were directly neighboring each other, and most of them had functions associated with stress responses.

We next analyzed protein gains and losses in the lineages leading to almond and peach, as inferred from the analysis of the gene trees in the phylome. When we analyzed the almond phylome, a total of 1471 proteins were gained in almond and 1146 were lost in peach. For the peach phylome we found that 1984 proteins were gained in peach and 3157 were lost in almond. Functional analysis shows that the proteins lost in almond with respect to peach are enriched in functions related to serine‐type endopeptidase inhibitor activity, nutrient reservoir activity, lipid transport, response to auxin, oxidation–reduction process and ion transport. Conversely, genes lost in peach with respect to almond are mainly enriched in functions related to transferase activity, transcription and ATP synthesis‐coupled proton transport (Table S7).

### Variability of almond cultivars

#### Read mapping rate, depth and genome coverage

Alignment of the 919 019 814 trimmed reads from the 10 re‐sequenced almond cultivars (Table S8) to our reference assembly resulted in mapping of 825 914 441 ‘clean’ reads (after removal of unmapped reads, PCR duplicates and reads with a mapping quality < 10) which corresponds to an average mapping rate of 89.6% (Table S9). Nonpareil and Vivot, and Falsa Barese and Genco showed the highest (94%) and lowest (79%) mapping rate, respectively (Table S9). Regarding sample depth and genome coverage, an average read depth of 39.7 was detected for the 10 cultivars, whereas 96.7% of the assembly was covered by the re‐sequencing data on average. Marcona and Falsa Barese had the highest (51.4×) and lowest (27.7×) read depth, respectively (Table S9).

#### Variant calling and phylogenetic analysis

Genetic variability analysis resulted in the detection of 2 253 377 variants, of which 2 203 582 (87%) were SNPs and 330 795 (13%) were insertions/deletions (indels). Genome‐wide distribution of SNPs and indels can be seen in Table S10. Nonpareil had the highest number of SNPs and indels, 1 072 759 and 142 142, respectively, whereas Ripon had the lowest number of SNPs and indels, 827 397 and 94 070, respectively. Average SNP density was calculated as 6.2 SNPs per kb, whereas the average heterozygosity for the 10 cultivars was 0.44% (Table S11). Our calculations of SNP density and heterozygosity for almond are lower than those recently published (a SNP density of 19.1 and heterozygosity of 0.69; Yu *et al.*, 2001). This discrepancy could be attributable to the use of the peach genome as a reference sequence during the variant calling in the published study, a varietal panel of larger genetic diversity or different filters and tools used for variant calling.

A graphical representation of SNP and indel distribution in windows of 100 kbp showed a similar profile for most of the almond cultivars analyzed (Figure S6). Nevertheless, lower overall variant density was observed in certain cultivars such as Genco, Falsa Barese and Ripon, which we attributed to the lower number of reads mapped in these genotypes.

An analysis of deletions in the collection of almond cultivars is presented in Table S12. For small deletions (1–50 bp), numbers were about half of those estimated for indels in Table S10 (about 60 000 versus about 120 000 per cultivar), as expected considering that only deletions and not insertions are considered. On average, 27% of these deletions overlapped with TEs. Considering large deletions (>50 bp), we detected 1219 unique events, the majority of which fell within the range 51–500 bp, with an average number of 88 deletions per cultivar. Marcona had the largest number of deletions (219) whereas Ripon had the lowest number (12) (Table S12). Five hundred and eighty‐eight large deletions (48.2% of the total) overlapped with TEs, with Nonpareil and Ripon showing nearly 60% of overlap with TEs and Cristomorto with the lowest percentage of overlap (38.8%). For deletions larger than 500 bp, almost all the events were found to overlap with TEs (92.6% in the range 501–10 000 and 100% in the range 10 001–50 000; Table S12, Figure S7).

A SNP‐based phylogenetic analysis grouped the almond cultivars into two main clades where the first clade contained Cristomorto, Falsa Barese and Genco while the second clade is split into two subclades, the first containing Aï, Belle d’Aurons, Nonpareil and Ripon and the second Desmayo largueta, Marcona and Vivot (Figure S8). This phylogeny is in agreement with the geographical origin of the analyzed cultivars, grouping the Italian cultivars, the French and US cultivars and finally the Spanish cultivars in the same clade. The fact that French and US cultivars are clustered together agrees with the known origin of US materials coming from French imported accessions (Kester *et al.*, 2015).

#### Indel variants between peach and almond and their relationship to TE sequences

To assess the structural variability between almond and peach genomes we aligned almond genome contigs to the peach reference genome. A total of 92.96% of the Texas almond assembly could be aligned to the Lovell reference peach genome sequence with an average identity of 95.59%, which increased to 97.99% (20 SNPs per kb) when only regions that align 1:1 are considered. We detected a total of 20 418 indel variants accounting for 18 Mb of sequence, equivalent to 8% of the almond genome (Table S13).

Re‐sequencing data from the peach cv. Earlygold were compared with the almond reference genome and deletions were identified, as previously done with almond cultivar re‐sequencing data (Table S12). The average number of deletions in almond cultivars compared with the almond reference sequence was 62 238, whereas in Earlygold this figure was more than double (126 137) (Table S12). However, when considering only deletions larger than 50 bp, peach had almost 12 times more (1436 versus 120) than almond.

### Transposable element landscape

**Figure 2:**
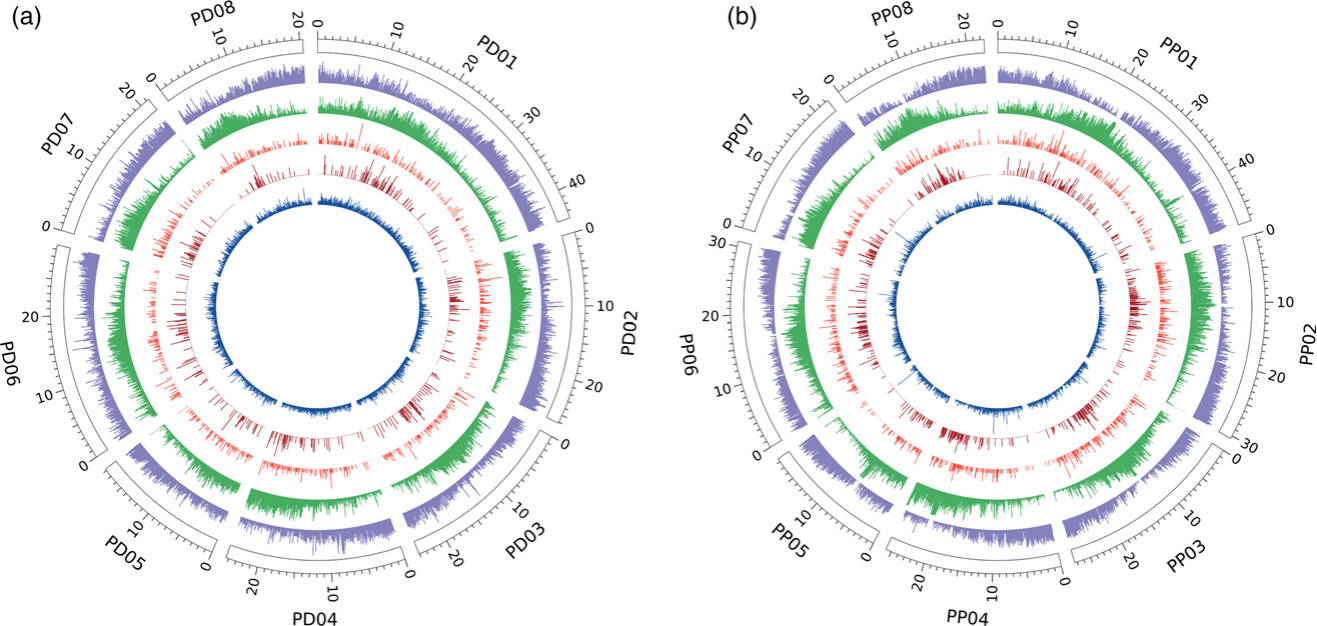
Distribution of gene and transposable element (TE) abundance. Distribution of gene and TE abundance along *Prunus dulcis* (a) and *Prunus persica* (b) chromosomes. Outer to inner tracks represent the coverage per 100 kb of genes, TEs, Copia long terminal repeat (LTR) retrotransposons, Gypsy LTR retrotransposons, and miniature inverted‐repeat transposable elements. The chromosome scale is in Mbp.

Using the REPET pipeline we annotated 38.21% of the almond genome as TE‐related sequences (Tables S14 and S15). The distribution of TEs along almond pseudochromosomes shows an inverse correlation with respect to the gene density, with TE‐rich regions showing low gene density per chromosome, coinciding with pericentromeric regions, and lower TE densities in the gene‐rich chromosomal arms (Figure 2a). The almond TE landscape was compared with that of peach. For that purpose, we annotated peach TEs with the same strategy and found very similar results: 37.60% of TE content (Tables S14 and S15) and a comparable TE and gene distribution to that of almond chromosomes (Figure 2b).

In addition to the general TE annotation, we performed a dedicated annotation of the long terminal repeat (LTR) retrotransposons and miniature inverted‐repeat transposable elements (MITEs) in the almond and peach genomes. A conservative search for LTR retrotransposons with a well‐preserved structure (i.e. presence of LTRs and coding capacity for retrotransposon‐related proteins) resulted in the annotation of approximately 2200 elements in both almond and peach (Table S16). Whenever possible, these elements were classified as Copia or Gypsy (i.e. when coding regions for both integrase and reverse transcriptase were detected, which allowed us to classify them) or remained as unclassified LTR retrotransposons. Although the content of these elements was similar in both genomes, the number of LTR retrotransposons that remained unclassified in almond was slightly higher. The distribution of the almond and peach retrotransposons along chromosomes is also highly similar, with Gypsy elements showing a tendency to concentrate in a central region of chromosomes, probably coinciding with the centromeric regions, whereas Copia elements are more evenly distributed (Figure 2). A conservative search for MITEs with well‐preserved Toll‐interleukin like regions (TIRs) rendered 10 460 MITEs in almond and 8738 MITEs in peach (Table S16). The distribution of these elements along chromosomes is similar in peach and almond and follows that of Copia LTR retrotransposons (Figure 2).

### The LTR retrotransposon dynamics in almond and peach

**Figure 3:**
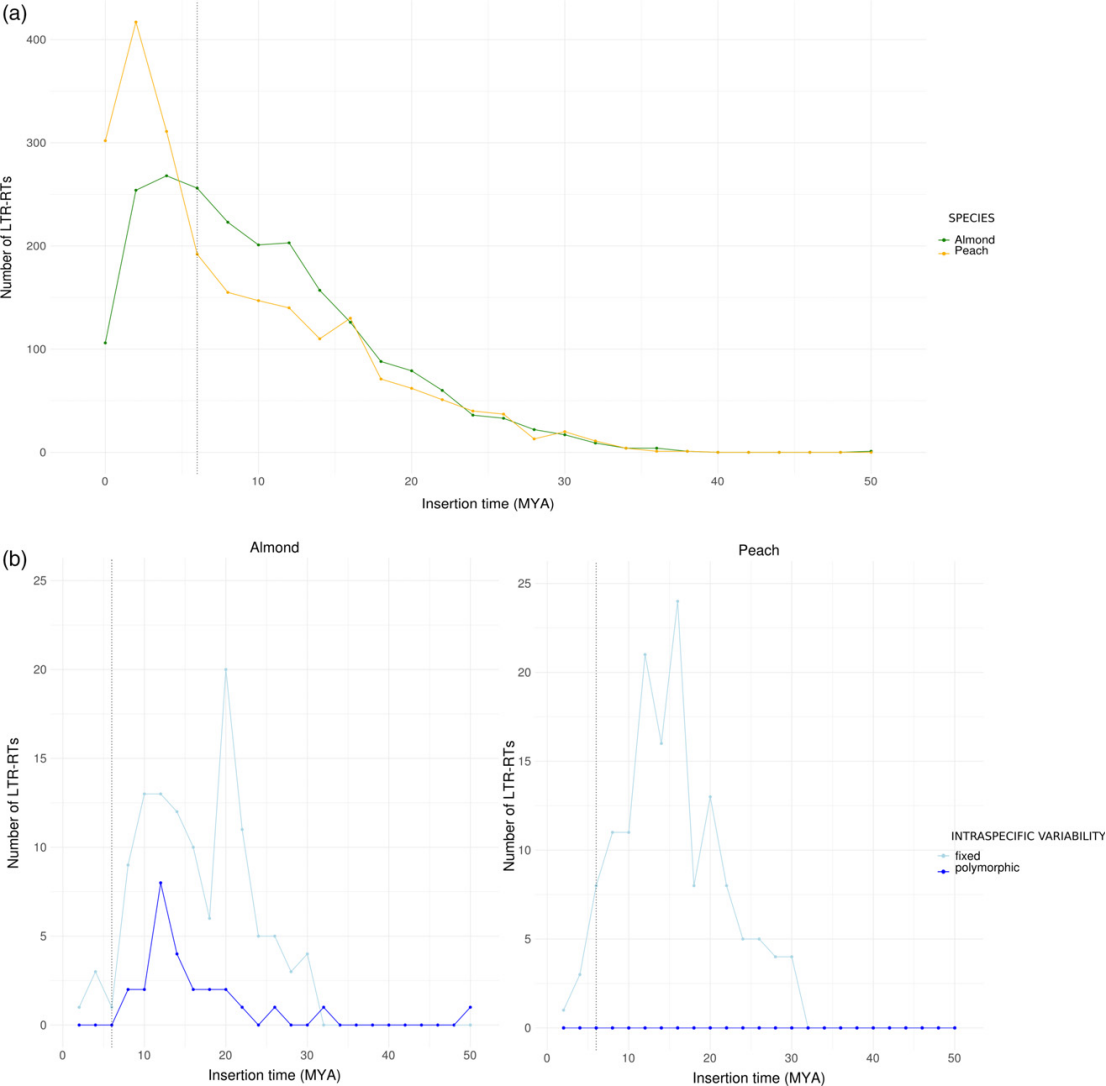
Dynamics of long terminal repeat (LTR) retrotransposons in peach and almond. (a) Insertion time of complete LTR retrotransposons in *Prunus dulcis* and *Prunus persica*. (b) Insertion time (MYA, million years ago) of polymorphic and fixed orthologous LTR retrotransposons in almond (left) and peach (right).

To gain insight into the evolution of almond and peach LTR retrotransposons, we grouped all almond and peach elements into clusters showing sequence identity higher than 80% along more than 80% of their length. Most of the elements (66.2%) were grouped into clusters of at least two elements. Two hundred and fifty‐nine clusters (81%) were mixed clusters and contained 93% of the almond and peach clustered elements. An analysis of the insertion times of these LTR retrotransposons shows that the number of recent (≤5 Mya) LTR retrotransposons is clearly higher in peach than in almond (Figure 3a). An analysis of the insertion time distribution of individual clusters within LTR retrotransposon families (belonging to Gypsy and Copia superfamilies, or that are unclassified), shows that many of them contain insertions that are younger in peach that in almond (Figure S9), suggesting that peach has experienced higher LTR retrotransposon activity after the evolutionary split of these two species.

To further understand LTR retrotransposon dynamics we analyzed the prevalence in the species of the LTR retrotransposon insertions found in peach and almond reference genomes by analyzing re‐sequencing data from 10 peach and 10 almond cultivars. This analysis shows that the LTR retrotransposon insertions are frequently polymorphic among almond cultivars whereas they often appear fixed in peach. An analysis of the insertion time distribution of fixed and polymorphic LTR retrotransposons in both species shows that whereas an important number of LTR retrotransposon insertions older than the estimated speciation time are polymorphic in almond, peach contains very few old polymorphic insertions, suggesting that they were lost in this species (Figure S10).

As all these analyses may be somewhat biased by a different degree of assembly of peach and almond genomes, we performed a detailed analysis of the LTR retrotransposon insertions comparing orthologous loci in both species. We were able to unambiguously identify the orthologous locus for 1155 full‐length LTR retrotransposon peach insertions and 1134 almond insertions (around 51% of the insertions in both species). These correspond to 142 insertions found intact in both species (conserved insertions), 592 and 440 specific insertions in peach and almond, respectively, 422 peach insertions partially deleted or rearranged in almond and 562 almond insertions partially deleted or rearranged in peach. An analysis of the ages of these LTR retrotransposon insertions belonging to the different categories showed that, as expected, the majority of the specific insertions in both genomes were younger than the estimated speciation date. The almond genome contains a larger fraction of specific insertions that are older, which probably corresponds to elements that were polymorphic in the ancestor and that were subsequently lost in peach. Also as expected, the vast majority of the conserved insertions were older than the estimated speciation time of peach and almond (Figure S11). In addition, analysis of the presence of these insertions in peach and almond cultivars using Pindel (see Experimental Procedures) showed that while all insertions are fixed in peach an important fraction is polymorphic in almond (Figure 3b).

### Transposon‐induced variability in peach and almond traits

The almond fruit resembles that of peach and other *Prunus* species, the major differences being that in almond the mesocarp does not develop to produce the fleshy tissue typical of other *Prunus* fruit crops, and that the almond seed does not accumulate the high levels of the cyanogenic diglucoside amygdalin that renders the seeds of peach and other *Prunus* species bitter and toxic. In order to shed light on the genetic differences underlying these phenotypic differences we compared the genomic regions containing the genes known to determine the expression of these characters in both species.

**Figure 4:**
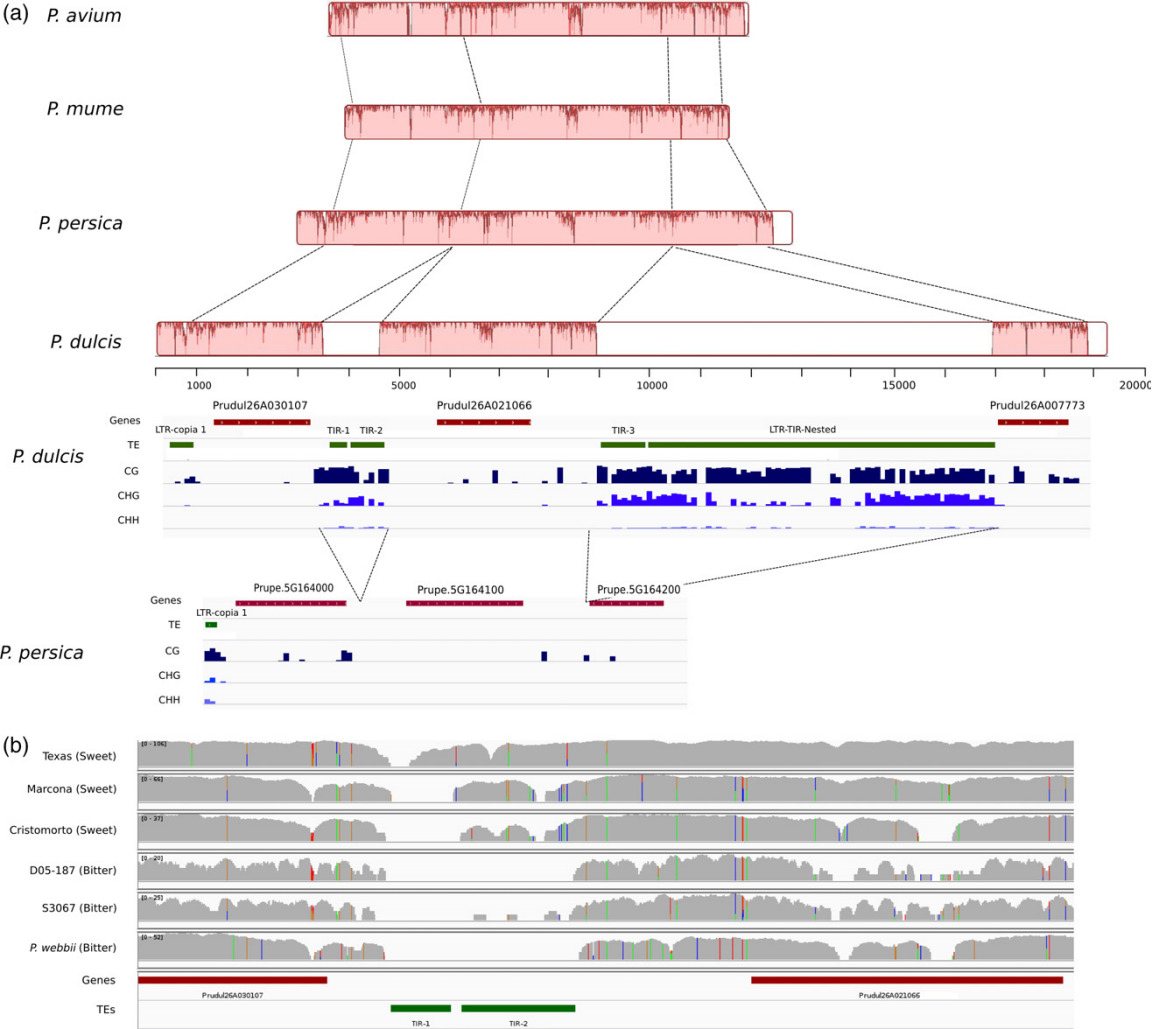
Analysis of the locus of the CYP71AN24 gene in almond varieties and related *Prunus* species. (a) Nucleotide conservation of the CYP71AN24 region between *Prunus avium*, *Prunus mume*, *Prunus persica* and *Prunus dulcis* based on a Mauve multiple alignment (physical distance scale is in bp). White boxes represent inserted regions in *P. dulcis*. The Integrative Genomics Viewer (IGV) tracks of the gene and transposable element (TE) annotations of *P. dulcis* and *P. persica* along with their DNA methylation levels in the three different contexts (CG, CHG and CHH) are shown below. (b) The IGV spnapshot of the region containing the *CYP71AN24* gene and the polymorphic TE insertions displaying the coverages of mapped DNA‐seq reads from re‐sequencing data of sweet‐ and bitter‐kernel *P. dulcis* varieties, as well as from that of the closely related *Prunus webbii.*

It has been recently shown that the sweet almond phenotype is due to the reduced expression of the genes encoding two cytochrome P450 enzymes catalyzing the first steps of amygdalin biosynthesis in sweet almond varieties compared with bitter almond varieties (Thodberg *et al.*, 2011). It has also been shown that this reduced expression is not related to differences in the gene sequence, which points to a difference in the regulation of the expression of those genes (Thodberg *et al.*, 2011). We compared the sequence of one of these almond genes, *CYP71AN24*, with its homologs in peach, sweet cherry and *P. mume* and found that it is highly conserved. However, *CYP71AN24* is flanked by several almond‐specific TE insertions, and in particular two MITE insertions in its proximal upstream region (Figure 4a). A preliminary analysis of the methylation of this region shows that the almond‐specific TE insertions flanking the *CYP71AN24* gene are highly methylated. The insertion of TEs in the proximal upstream region of the *CYP71AN24* gene may have affected, directly or indirectly, its expression due to its high methylation. In addition, the presence of the TEs also correlates with a much higher methylation of this gene in almond compared with peach (11.6% methylation at CG and 0.1% methylation at CHG in almond versus 0.1% methylation at CG and 0.02% at CHG in peach), which may be the result of methylation spreading from the TEs. An analysis of the structure of this locus in *Prunus webbii*, a wild species closely related to almond which produces bitter seeds, and in two sweet almond cultivars (Cristomorto and Marcona) and two almond cultivars producing bitter seeds (D05‐187 and S3067) performed by mapping the re‐sequencing data of these genomes to the almond reference genome, shows that the presence of the TE insertions (and in particular that of the MITE named TIR2), correlates with the sweet versus bitter seed phenotype (Figure 4b). Moreover, an article published after the submission of our research reports a strong association between the sweet almond phenotype and a point mutation in a gene (*bHLH2*) encoding for a bHLH transcription factor that renders it unable to bind to the promoter of one of these genes, *CYP71AN24* (Sánchez‐Pérez *et al.*, 2019). An analysis of the sequence of the genome produced here, the sweet almond Texas, as well as the mentioned sweet and bitter accessions and *P. webbii* shows that this mutation is absent in Texas and, on the contrary, it is present in *P. webbii*, being as expected in the rest of the cases. Additionally, Sánchez‐Pérez *et al. *(2019) reported that one of the sweet almond cultivars they analyzed (Atocha), did not have the same mutation in *bHLH2* but had another one in the neighborhood.

The analysis of model plant species, such as Arabidopsis and tomato, has shown that the development of the fruit is the result of the combined action of genes involved in meristem organization, floral development and fruit cell proliferation and expansion. We selected 97 genes (Table S17) that belong to gene families involved in these processes, including *WUSCHEL* (*WUS*) and *CLAVATA* (*CLV*) genes whose mutants lead to larger fruits in tomato and genes known to determine fruit shape (Rodriguez *et al.*, 2011). We have analyzed the structure of these genes in peach and almond and found that six of them present differential TE insertions within the genes in their proximal (less than 1000 nucleotides) upstream region that probably contains their promoter. These species‐specific TE insertions are all highly methylated and may have altered their expression (Table S18). In addition to the potential mutation of transcriptional regulatory elements, MITE insertions could have provided other transcription factor‐binding sites (TFBS), as MITEs have been shown to frequently amplify and mobilize TFBS in plants (Morata *et al.*, 2018).

## Discussion

Using a hybrid strategy based on short and long‐read DNA sequences and the information provided by existing linkage maps we have assembled the highly heterozygous genome of almond cv. Texas into a rather complete, contiguous and low‐redundant assembly with eight pseudomolecules corresponding to the eight chromosomes and comprising 227.6 Mbp of sequence. Annotation of this genome has resulted in the identification of 27 969 protein‐coding genes and 6747 non‐coding transcripts. The assembled sequence is highly syntenic with the genome sequence of peach (Verde *et al.*, 2017), as was expected considering previous information on the close genetic similarity between these two species (Dirlewanger *et al.*, 2004).

Based on molecular data and the use of fossil records of a diverse sample of 17 plant species we estimated the divergence times of *P. dulcis* with respect to other sequenced *Prunus*. Our estimate of 5.88 Mya for the divergence of peach and almond from a common ancestor is similar to that of recent molecular evidence that places this figure between 5.0 and 8.0 Mya (Yu *et al.*, 2001; Velasco *et al.*, 2014; Delplancke *et al.*, 2016). This is compatible with the separation of the ancestral species by the uplift of the Central Asian Massif in two subpopulations that faced completely different environments: one (almond and its close relatives) in the arid steppes of central and western Asia and the other (peach) in the subtropical climate of southwestern China, close to where the first fossil endocarps were found dated at 2.6 Mya (Su *et al.*, 2015).

In agreement with results from earlier studies of other sequenced diploid *Prunus* genomes (Shirasawa *et al.*, 2017; Verde *et al.*, 2017; Zhang *et al.*, 2017; Baek *et al.*, 2018) we have not found evidence for any recent whole‐genome duplication of almond. Analysis of duplicated gene sequences indicates a parallel gene expansion for all sequenced *Prunus* species genomes for genes involved in lignin biosynthesis, such as COMT and HCT. One of the distinctive aspects of *Prunus* is that its fruit is a drupe, characterized by the formation of a strongly lignified mesocarp (the ‘stone’), unlike most of its closest taxa that have follicetum and nuculanium as fruit types (Xiang *et al.*, 1990). These duplications may be at the origin of the formation of the stone, determine its characteristics and be crucial to understanding its evolution and possible modification, with important consequences for plant breeding, including the production of stoneless cultivars in *Prunus* fruit (Callahan *et al.*, 2015).

As we have shown from the comparison of the peach and almond reference genomes, as well as the analysis of the structural variants between both genomes, indel events seem to explain a substantial part of the divergence of peach and almond genomes from their common ancestor. In this study we show that most of such structural differences, particularly those of larger sizes, were related to TE sequences. The LTR retrotransposons and MITEs constitute the two most prevalent superfamilies of TEs in plants (Casacuberta and Santiago, 2003). While the proportion of the almond genome consisting of TEs (38%) was similar to that of other sequenced genomes of similar size, that is from 30% in peach (Verde *et al.*, 2013a,b) and 43–47% in other *Prunus* species (Shirasawa *et al.*, 2017; Zhang *et al.*, 2017; Baek *et al.*, 2018) and very similar chromosomal distributions of TEs were observed for almond and peach, detailed analysis of the dynamics of LTR retrotransposon evolution has revealed key aspects of the divergence of almond and peach genomes after their speciation. In short, almond has maintained more ancestral LTR retrotransposons, which are still in some cases polymorphic within the species, whereas peach has lost most polymorphic ancestral insertions but seems to have witnessed a higher level of recent retrotransposition activity. The contrasting data on the polymorphism of ancestral TEs between peach and almond may be explained by (i) the mating types (selfing in peach and outcrossing in almond) and (ii) differences in their recent history, with a strong reduction of population size in peach prior to its recent expansion as a cultivated species 2000 years ago (Velasco *et al.*, 2014), while almond population sizes remained higher (Yu *et al.*, 2001). Further analyses should help to clarify this in the future. In any case, the results presented here suggest that LTR retrotransposons, and in general TEs, may explain an important fraction of the interspecific variability between peach and almond, as well as the intraspecific variability of both species.

Long terminal repeat retrotransposons are at the origin of somatic mutations in plant species, some of which have high agricultural value (Foster and Aranzana, 2018). Only in peach, white versus yellow fruit color (Falchi *et al.*, 2013), hairy versus glabrous fruit (Vendramin *et al.*, 2013) and stonyhard versus melting flesh texture (Tatsuki *et al.*, 2018) are caused by the action of transposon movement. The MITE insertions have also been linked to crop traits, such as sex determination in melon (Martin *et al.*, 2009) or a drought tolerance phenotype in maize (Mao *et al.*, 2015). The analysis of almond × peach interspecific progeny identified 11 Mendelian genes explaining the inheritance of some key agronomic characters: one of them, responsible for the formation of the thick mesocarp that constitutes the peach flesh, and another, conferring juiciness to the fleshy mesocarp, represent major contributions to the difference between almond and peach fruits (Donoso *et al.*, 2016). Our results suggest that TEs could be responsible for some of the genomic changes at the origin of the agronomic traits that distinguish peach from almond, such as mesocarp development and bitterness of the kernel. For one of them, sweet versus bitter kernel, which is essential for the domestication of the almond, we show here that the sweet almond phenotype correlates with the presence of TE insertions surrounding the gene *CYP71AN24*. This gene is involved in the synthesis of one of the key enzymes of the amygdalin pathway (cytochrome P450), and it has been proposed that its reduced expression, together with the lack of expression of *CYP79D16*, results in the sweet kernel trait (Thodberg *et al.*, 2011). It has been recently shown that the sweet kernel phenotype is closely associated with a point mutation in a bHLH transcription factor (Sánchez‐Pérez *et al.*, 2019). These authors identified a sweet almond cultivar that did not have the mutation, and we found two more examples (Texas and *P. webbii*) with unexpected genotypes. This shows that, although there is a good correlation of this mutation in the bHLH transcription factor gene and the sweet almond phenotype (Sánchez‐Pérez *et al.*, 2019), this association may not be perfect, suggesting that other mechanisms to avoid the activation of *CYP71AN24* could contribute to the sweet kernel phenotype. We propose here that the presence of highly methylated TE insertions in the close proximity of *CYP71AN24* could help to ensure the low expression of this gene in the seed tegument of sweet almonds. Additionally, TEs may be at the origin of other important traits in almond. We identified six genes related to fruit flesh formation that also contain highly methylated TEs in their upstream region in either almond or peach. Although we have not been able to associate any of these genes with the positions of major genes or quantitative trait loci described so far in these species, they are clear candidates for further studies.

In summary, peach and almond diverged less than 6 Mya, which for a perennial species with a long intergenerational period of around 10 years, is a short evolutionary time for sequence divergence. Indeed, our results show that the genomes of peach and almond are highly similar and only show a mean of 20 nucleotide substitutions per kb. Our results here show that this relatively low sequence divergence is accompanied by an important number of indels frequently resulting from specific TE insertions, which in some cases could be at the origin of important almond characteristic traits. The activity of TEs is not constant through evolution, alternating between quiescent periods and transposition bursts, which, as our results suggest here for almond, could allow for a rapid phenotypic diversification between closely related species.

The genome sequence of the almond will accelerate genetic research and facilitate breeding of this species by providing useful information on genes and markers with an unprecedented level of detail, as is the case in other *Prunus* species (Aranzana *et al.*, 2019). It can also help further our understanding of the evolution and domestication of closely related crop species that share a slower rate of evolution due to their long intergeneration period, and may enable the detection of modes and aspects of evolution that could be different or otherwise difficult to identify in herbaceous crops (Gaut, 2015).

## Experimental procedures

### Extraction of DNA, sequencing and *k*‐mer analysis

Fresh young leaves of the Texas almond were ground in liquid nitrogen to a fine powder, and 100 mg of ground leaves was used for DNA extraction using the DNeasy® Plant Mini Kit (Qiagen, https://www.qiagen.com/) according to the manufacturer’s instructions. The same method was employed for the extraction of 10 additional almond cultivars (Aï, Belle d’Aurons, Cristomorto, Desmayo largueta, Falsa Barese, Genco, Marcona, Nonpareil, Ripon and Vivot), eleven peach cultivars (Armking, Belbinette, BigTop, Blanvio, Catherine, Earlygold, Flatmoon, Nectalady, Platurno, Sweetdream and Tiffany) and one accession of the wild almond *P. webbii* (R755). For sequencing with an Oxford Nanopore Technologies (ONT; https://nanoporetech.com/) MinION sequencer, high‐molecular‐weight DNA from Texas almond was extracted with the method described by Mayjonade *et al.* (2016).

Whole‐genome shotgun sequencing was performed for Texas DNA using the Illumina HiSeq2000 sequencing instrument. The standard Illumina protocol with minor modifications was followed for the creation of short‐insert paired‐end (PE) libraries (Illumina Inc., cat. no. PE‐930‐1001, https://www.illumina.com/). In brief, three libraries were generated from > 2.0 μg of genomic DNA each. For one library the DNA was amplified by PCR while for the other two libraries (and the majority of the sequence) the DNA was not amplified in order to reduce GC bias. Then the DNA was sheared on a Covaris™ E220, the fragmented DNA was size selected on an agarose gel to obtain three PE libraries with incremental insert sizes of 263, 317 and 354 bp. The fragments were end‐repaired, adenylated and ligated to Illumina indexed PE adaptors. The PE libraries were run on the of Illumina HiSeq2000 platform in 2 × 101 PE mode according to standard Illumina operation procedures. Primary data analysis was carried out with the standard Illumina pipeline (HCS 2.0.12.0, RTA 1.17.21.3). A total of 97 Gb of raw sequence (>350× coverage) was produced. Post‐processing of sequence reads involved detection and trimming of Illumina adapter sequences with cutadapt, quality trimming with trim_galore and PE overlap detection and merging with FLASH. Mate pair (MP) libraries of Texas DNA (fragment sizes 3.1 and 5.2 kb) were constructed according to the Nextera Mate Pair Preparation protocol, which leaves a linker of known sequence at the junction. The resulting libraries were run on the HiSeq2000 platform in 2 × 101 bp read length runs. Post‐processing of sequence reads involved detection and trimming of the Nextera linker sequence with cutadapt, and quality trimming with trim_galore.

Fosmid pools from Texas almond were used to prepare 2D genomic libraries using the Ligation Sequencing Kit SQK‐MAP005/006 or SQK‐NSK007. The sequencing run was performed on an R7.3 chemistry FLO‐MAP103 or R.9 FLO‐MIN104 flow cell (ONT) according to the manufacturer’s recommendations. Genomic DNA from Texas almond was also used to prepare 1D and 1D2 genomic libraries using the Ligation Sequencing Kits SQK‐LSK108 and SQK‐LSK308, respectively. The sequencing run time on FLO‐MIN106 or FLO‐MIN107 flow cells using the MinION/MKI Pk.1 instruments (ONT) was 48 h. The quality parameters of the sequencing runs were further monitored by the MinKNOW platform, while the run was base‐called using the Metrichor agent (https://metrichor.com) in real time.

For the almond cultivars other than Texas and the peach and *P. webbii* accessions, we developed PE libraries of fragment size 300 bp and sequenced them with Illumina as described in the previous paragraph.

Jellyfish v.2.2.6 (Marçais and Kingsford, 2011) was run on the PE300 library (insert size 317 bp, 2 × 100‐nucleotide reads) with the canonical *k*‐mer (‐C) option and a mer‐size of 21. GenomeScope (Vurture *et al.*, 2006) was then used to analyze the resulting *k*‐mer distribution. The ONT reads were error‐corrected using Canu (v1.5) (Koren *et al.*, 2017). Corrected trimmed reads were used for hybrid assembly, scaffolding and assembly correction.

### Genome assembly

Short‐read whole‐genome sequence (WGS) assembly of 100 bp PE read Illumina libraries with AbySS v.1.3.6 (Simpson *et al.* 2009) resulted in a fragmented assembly (N50 = 4867 bp) with inflated genome size (512 Mbp). Heterozygosity and repeats were clearly going to be a problem. Fosmid‐pool sequencing (150/250 nucleotide PE reads) and assembly combining whole‐genome sequencing PE and MP reads as previously described (Abascal *et al.*, 2016; Cruz *et al.*, 2016) increased contiguity (N50 = 142 kb) and reduced the assembly size (238 Mbp); however, the resulting assembly exhibited high levels of discordance with the available genetic maps, as well as with the peach assembly (v.2.0.1). The final strategy, combining long‐read ONT sequencing and whole‐genome sequencing PE reads, resulted in the best balance of contiguity and concordance with the genetic map.

The hybrid assembler MaSuRCA v.3.2.3 (Zimin *et al.*, 2013) was run with default parameters (no linking mates; Celera assembly of super‐reads). The input was the two PE Illumina libraries of insert sizes 317 and 354 bp for a total of 285× coverage (Table S1) and the self‐corrected ONT reads for a total of about 20× coverage.

Redundans v.0.13 (Pryszcz and Gabaldón, 2016) was run on the non‐deduplicated output of MaSuRCA (the step 9‐terminator genome.ctg.fasta file totaling 470 Mb with N50 = 53 kb) using the –longreads option for scaffolding, reducing the size of the assembly to 248 211 Mb of which was scaffolded further with the ONT reads.

A first round of corrections to the assembly was carried out using consistency with nanopore data as the main criteria. ONT reads were mapped to the assembly with NGM‐LR v.0.2.6 (Sedlazeck *et al.*, 2018), the assembly was broken at regions of zero coverage and then re‐scaffolded with SSPACE‐LongRead v.1.1 (Boetzer and Pirovano, 2014). A second round of corrections was made utilizing collinearity with the T×E genetic F_2_ and BC1 (to Earlygold) linkage maps (Donoso *et al.*, 2015) as the main criterion, with break points guided by synteny with the peach genome and coverage of ONT reads. Peach transcripts (annotation Pp2.01a) were mapped to the almond assembly with GMAP v.2014‐12‐23 (Wu and Watanabe, 2017). Marker sequences were mapped with BWA mem, keeping those mappings with mapping quality ≥ 20 and identity ≥ 90%. The broken assembly was again scaffolded with SSPACE‐LR. The assembly at this stage had a contig N50 of 99 kb and scaffold N50 of 151 kb.

A third round of corrections was performed with improvements in mapping and break detection. First, peach transcripts were mapped only in the sense direction, and second, discrepant marker mappings were screened for mapping artifacts. Moreover, Sniffles v.1.0.11 (Sedlazeck *et al.*, 2018) was used for detection of structural variants. Additional breaks were made and duplicate sequences were also detected. In the end, we were able to merge 30 Mb with minimus2 from AMOS v.3.1.0 (Sommer *et al.*, 2007; Treangen *et al.*, 2010) and any remaining duplicate sequence (>99% identical, >5 kb) was manually reviewed. Overlapping regions were joined into new longer contigs using nanopore read mappings to confirm new joins. Also at this stage, the putative chloroplast sequence was identified by coverage and homology and set aside.

Finally, the assembly was anchored to pseudomolecules using both the T×E genetic map and synteny with the peach genome using ALLMAPS (jcvi‐0.7.3) (Tang *et al.*, 2018), with more weight given to the map marker order. Remaining conflicts were resolved manually. ALLMAPS uses a genetic algorithm for placing and orienting scaffolds, and sometimes it does not converge completely on the optimal solution, even with a large number of generations. Thus, we had to manually review and fix the order and orientation of some scaffolds which still exhibited discordance with either the genetic map or synteny with peach. Further improvement to the assembly was made by joining adjacent scaffolds if they could be linked together with split nanopore read mappings. A few additional overlaps were also detected in this fashion and longer contigs were constructed.

Assembly completeness was estimated in two ways. First, gene completeness was determined by running BUSCO v.3.0.2 (Simão *et al.*, 2015) using the embryophyta_odb9 database comprising 1440 single‐copy plant orthologous groups (BUSCOs). Second, a pairwise comparison of *k*‐mers present in both input reads and the assembly was performed using KAT (Mapleson *et al.*, 2017) using all whole‐genome sequencing PE Illumina reads and a *k*‐mer length of 27 (Figure S12).

### Comparison of the *P. dulcis* anchored assembly to the linkage map and peach v.2.0 a1 genome sequence

The almond assembly was compared with the T×E linkage map that contains 1833 SNP markers (Donoso *et al.*, 2015). Markers were mapped onto the almond pseudomolecule‐based assembly using BLAST and coordinate data of both almond and peach were used as input in MapChart software (Voorrips, 2017) for representing graphically the comparison between the two species. The genetic and physical distances of SNP markers from the T×E population were used for calculating the recombination rate across the pseudomolecules of the almond assembly.

The peach genome sequence and annotation data were downloaded from the GDR (ftp://ftp.bioinfo.wsu.edu/species/Prunus_persica/Prunus_persica-genome.v2.0.a1/). Synteny between almond with peach genomes was assessed using SyMap software v.4.2 (Soderlund *et al.*, 2007) with default parameters, except that the ‘min dot’ parameter was set to 25.

### Annotation

The *P. dulcis* genome assembly was annotated by combining transcript alignments, protein alignments and *ab initio* gene predictions. A flowchart of the annotation process is shown in Figure S13. Scripts are available at https://github.com/jesgomez/annotation_pipeline.

First, almond RNA‐seq reads were downloaded from NCBI with the accession number SRR1251980 and aligned to the genome with STAR (v.2.5.3a) (Dobin *et al.*, 2013). Transcript models were subsequently generated using Stringtie (v.1.0.4) (Pertea *et al.*, 2015) and, along with the *P. persica* transcriptome (annotation Pp2.0a) and 4509 almond expressed sequence tags downloaded from NCBI on July 2015, were assembled into a non‐redundant set by PASA (v.2.3.3) (Haas *et al.*, 2008). The TransDecoder program, which is part of the PASA package, was run on the PASA assemblies to detect coding regions in the transcripts. Second, the complete Rosaceae proteome was downloaded from Uniprot on July 2015 and aligned to the genome using Exonerate (v.2.4.7) (Slater and Birney, 2005). Third, *ab initio* gene predictions were performed on the repeat masked pdulcis26 assembly with three different programs: GeneID v.1.4 (Alioto *et al.*, 2018), Augustus v.3.2.3 (Stanke *et al.*, 2015) and GeneMark‐ES v.2.3e (Lomsadze *et al.*, 2014) with and without incorporating evidence from the RNA‐seq data. Finally, all the data were combined into consensus coding sequence models using EvidenceModeler‐1.1.1 (EVM) (Haas *et al.*, 2008). Additionally, untranslated regions and alternative splicing forms were annotated through two rounds of PASA annotation updates.

Non‐coding RNAs were annotated as follows: first, the program cmsearch v.1.1 (Cui *et al.*, 2016) from the INFERNAL package (Nawrocki and Eddy, 2013) was run against the RFAM (Nawrocki *et al.*, 2015) database of RNA families (v.12.0). Also, tRNAscan‐SE v.1.23 (Lowe, 1997) was run to detect the transfer RNA genes present in the genome assembly. To annotate long non‐coding RNAs (lncRNAs) we first selected PASA assemblies that had not been included in the annotation of protein‐coding genes. Those longer than 200 bp and whose length was not covered to at least 80% by a small ncRNA were incorporated into the ncRNA annotation as lncRNAs. The resulting transcripts were clustered into genes using shared splice sites or significant sequence overlap as criteria for designation as the same gene.

#### Functional annotation

Functional annotation was performed by integrating several data sources to infer protein function based on sequence similarity to annotated sequences or/and the presence of particular domains and sequence motifs. We used the InterPro (Hunter *et al.*, 2012), KEGG (Kanehisa *et al.*, 2012), signalP (Petersen *et al.*, 2011) and NCBI CDsearch (Marchler‐Bauer *et al.*, 2011) databases. InterProScan v.5.19‐58 (Zdobnov and Apweiler, 2012) was used to scan though all available InterPro databases, including PANTHER, Pfam, TIGRFAM, HAMAP and SUPERFAMILY. Initial sequence similarity search was determined using BLASTP v.2.6.0 + against the NCBI non‐redundant (NR) collection of protein sequences (release 2018‐08). KEGG orthology (KO) groups were assigned by the KEGG Automatic Annotation Server (KAAS) (Moriya *et al.*, 2007) using the bi‐directional best hit (BBH) method against a representative gene set from 27 different species, which includes a core set of species for gene annotation and additional plant species from the Rosaceae family. The KO identifiers were then used to retrieve the relevant KEGG functional annotation using the KEGG REST‐based API service, KEGG release v.87.1.

To predict plant disease resistance genes, each protein was searched against a manually curated list of ‘reference’ R‐genes with the DRAGO pipeline (Sanseverino *et al.*, 2013). For each hit, classes were assigned based on combination of specific domains, such as TIR, nucleotide‐binding site (NBS), leucine‐rich region (LRR) or coiled‐coil domain. Putative transcription factor genes were predicted using the Plant Transcriptional Factor database (Jin *et al.*, 2017) v.4.0

#### Annotation and analysis of transposable elements in genome assemblies

The Ilumina PE reads corresponding to the re‐sequencing of the almond and peach cultivars described in section "DNA extraction, sequencing and K‐mer analysis" were trimmed with SKEWER (v.0.2.2, –mean‐quality 25, –min 35) and aligned to their respective reference genome with BWA‐aln/sampe^1^ (v.0.7.5, parameters: ‐t 6, ‐n 5, ‐o 1, ‐e 3) (Li and Durbin, 2009) and SAMTOOLS (v.0.1.18) (Li, 2011). Bam files were later submitted to the package PINDEL (v.0.2.5, parameters: ‐T 4, ‐x 5, ‐r false, ‐t false, ‐A 35) (Ye *et al.*, 2018) to identify deletions in samples. The TEs were annotated in *P. dulcis* and *P. persica* assemblies using TEdenovo and TEannot pipelines of the REPET package (Quesneville *et al.*, 2005; Flutre *et al.*, 2011) installed in the PiRATE virtual machine (Berthelier *et al.*, 2018). Classification of TEdenovo consensus sequences at the order level was done with PASTEC (Hoede *et al.*, 2014).

Annotation of TEs for masking purposes was done using RepeatMasker (http://www.repeatmasker.org/) with a reduced TE representative library. The TE representatives obtained using the TEdenovo pipeline library were screened for coding domains with hmmscan (HMMER 3.1b1, http://hmmer.org/) against the PFAM database (Finn *et al.*, 2016). The TE representatives containing regions potentially coding for known domains of non‐TE proteins usually found in multigene families (kinases, NB‐ARC, LRR, TIR) or with an N content higher than 30% or with length shorter than 200 nucleotides were discarded. Moreover, all TE representatives not categorized in one of the classical TE superfamilies (defined as ‘noCat’ by TEdenovo) were also removed. A total of 661 representatives were removed from the library. The final library contained 6898 TE representatives.

MITE‐hunter (Han and Wessler, 2010) was run to detect potential MITE families. In order to complete the annotation, the potential almond MITE families were combined with the *P. persica* family annotation available in the PMITE database (families carrying target site duplication) (Chen *et al.*, 2014). These sequences were grouped in clusters of 90% identity with cd‐hit (Fu *et al.*, 2012) to remove redundancy and produce a final library of family representatives. RepeatMasker (http://www.repeatmasker.org/) was run to annotate all regions having significant similarity to MITE families, and the results were filtered to retain only full‐length elements (consensus length ± 20%). The same pipeline was used to identify MITEs in the *P. persica* assembly.

Candidate LTR retrotransposons were predicted by running LTRharvest (Ellinghaus *et al.*, 2008) with default parameters. The internal conserved domains of these elements were identified using HMMER hmmscan (Johnson *et al.*, 2010) and only coding elements were retained for further analyses. Elements displaying either a single hit on the genome, more than 10% of gaps or more than 50% of tandem repeats were filtered out. Classification of the remaining elements (hereafter referred to as ‘coding LTR retrotransposons’) into Copia and Gypsy superfamilies was performed based on the order of the internal coding domains, as defined by Xiong and Eickbush (2006). Elements lacking one or more domains were tagged as ‘incomplete’.

The LTR regions of every coding element were extracted and aligned with MUSCLE (Edgar, 2004). The Kimura two‐parameter distance of every aligned LTR pair was calculated and used to estimate insertion ages following the approach described in (SanMiguel *et al.*, 1998), using a substitution rate of 10^−8^ nucleotides per site per year and a generation time of 10 years (Velasco *et al.*, 2014).

The flanking sequences (500 bp) of every coding LTR retrotransposon were extracted from *P. dulcis* and used as query for a BLASTn (Altschul *et al.*, 1990) search (cutoff E‐value < 10^–10^) against the *P. persica* assembly, and vice versa. Concordant mapped flanks were defined when both flanks of an element mapped in the same scaffold at a distance smaller than 25 kb. Every internal region between two concordant mapped flanks was aligned to the putative orthologous element using EMBOSS Needle (Rice *et al.*, 2000). The two elements were considered orthologous if they could be aligned over 80% of their length with at least 80% identity. To assess the orthology of MITE insertions the approach followed was as the one described for LTR retrotransposons except that the sequences flanking the insertions were mapped to the corresponding genome using BBmap (https://sourceforge.net/projects/bbmap/) instead of Blast.

In order to search for polymorphic LTR retrotransposon and MITE insertions within or close to genes we used BEDTools (v.2.27.0) (Quinlan and Hall, 2010). Only those TEs located within genes or at less than 1000 nucleotides upstream of a gene were kept.

### Analysis of DNA methylation in almond and peach

Genomic DNA (1.5–2 μg) from young leaves of *P. dulcis* (cv. Texas) and *P. persica* (cv. Earlygold) was spiked with unmethylated bacteriophage λ DNA (5 ng of λ DNA/μg of gDNA; Promega, https://www.promega.com/) and with methylated T7 phage DNA (5 ng of T7 DNA μg^–1^ of gDNA). The gDNA was sheared on a Covaris™ E220 and fragments of 150–300 bp were size‐selected using AMPure XP beads (Beckmann Coulter, Brea CA, USA). The libraries were constructed using the Kapa Library Preparation kit (Roche Kapa Biociences, Pleasanton CA, USA) for short‐insert paired‐end libraries for Illumina with some minor modifications. After ligation of the NEXTFLEX^®^ Bisulfite‐Seq Barcodes (Perkin Elmer, https://www.perkinelmer.com/) the library was treated with sodium bisulfite using the EpiTect Bisulfite Kit (Qiagen), following the manufacturer’s instructions for formalin‐fixed, paraffin‐embedded tissue samples. Two rounds of bisulfite conversion were performed to ensure a conversion rate of over 99%. Enrichment for adaptor‐ligated DNA was carried out through seven PCR cycles using KAPA HiFi Uracil + DNA Polymerase (Kapa Biosystems, https://www.kapabiosystems.com/). Library quality was monitored using the Agilent 2100 Bioanalyzer, and the library concentration was estimated using quantitative PCR with the library quantification kit from Roche Kapa Biosystems. Paired‐end DNA sequencing (2 × 101 + 8 bp) was then performed using the HiSeq2500 (Illumina) following the manufacturer’s protocol. Image analysis, base calling and quality scoring of the run were processed using the manufacturer’s software Real Time Analysis (RTA 1.18.66.3) and followed by generation of FASTQ sequence files. Raw reads were trimmed with TrimGalore! v.0.4.5 (http://www.bioinformatics.babraham.ac.uk/projects/trim_galore/). Low‐quality bases (Phred score < 20) were trimmed before adapter removal and reads with a length less than 20 were discarded. The total of trimmed reads was 82 073 678 and 94 040 426 in almond and peach, respectively. Trimmed reads of each species were mapped to their respective reference genome and methylation was analyzed using Bismark v.0.19.1 (Krueger and Andrews, 2011). The gene/TE methylation was analyzed with SeqMonk v.1.41 (http://www.bioinformatics.babraham.ac.uk/projects/seqmonk/). Only cytosine positions that had been sequenced at least three times were included.

### Analysis of the CYP71AN24 locus in almond cultivars and *Prunus*‐related species

A 2‐Mb region of the *P. dulcis* genome containing the *CYP71AN24* gene was compared with the corresponding genomic regions of *P. avium*, *P. mume*, *P. persica* using Mauve (Darling *et al.*, 2004). Re‐sequencing data from the *P. dulcis* cultivars Texas, Marcona, Cristomorto, D05‐187 (SRX245830) and S3067 (SRX245832), as well as from *P. webbii* (R755), were mapped to the almond reference genome using BWA aln/sampe (Li and Durbin, 2009).

### *Prunus dulcis* phylome reconstruction

The *P. dulcis* and *P. persica* phylomes, that is the complete collection of evolutionary histories of all encoded genes, were reconstructed using the PhylomeDB pipeline (Huerta‐Cepas *et al.*, 2011). In brief, for each protein‐coding gene in the almond and peach genome we searched for homologs (Smith–Waterman Blast search, *E*‐value cutoff < 1 × 10^–5^, minimum contiguous overlap over the query sequence cut‐off ≥ 50%) in a database containing the proteomes of 17 species with sequenced genomes representing most of the important plant families (Table S3). The most similar 150 homologs were aligned using three different programs, MUSCLE (Edgar, 2004), MAFFT (Katoh *et al.*, 2009) and KALIGN (Lassmann and Sonnhammer, 2005), in forward and reverse orientation. These six alignments were combined using M‐COFFEE (Wallace *et al.*, 2008) and trimmed with trimAl v.1.3 (Capella‐Gutiérrez *et al.*, 2009), using a consistency cut‐off of 0.16667 and a gap threshold of 0.1. Phylogenetic trees were built using a maximum likelihood approach as implemented in PhyML v.3.0 (Guindon and Gascuel, 2003) using the best fitting model among seven different ones (JTT, LG, WAG, Blosum62, MtREV, VT and Dayhoff). The model best fitting the data was determined by comparing the likelihoods estimated on an initial neighbor joining tree topology and using the Akaike information criterion. In all cases we used four rate categories and inferred the fraction of invariant positions and rate parameters from the data. Then, these phylomes were filtered to remove the gene trees that contain proteins associated with transposon‐related functional terms. All alignments and trees are available for browsing or download at PhylomeDB with the PhylomeID 406 (almond phylome) and 407 (peach phylome) (Huerta‐Cepas *et al.*, 2014) (http://www.phylomedb.org/).

#### Prediction of orthology and paralogy and detection of gene duplications

Orthology and paralogy relationships were predicted based on phylogenetic evidence from the almond and peach phylomes. We used ETE v.3 (Huerta‐Cepas *et al.*, 2010) to infer duplication and speciation relationships using a species overlap approach and a species overlap score of 0. In brief, the algorithm traverses the tree from the tip to the root and, for each node, evaluates whether the two daughter branches contain genes from the same species, in which case a duplication is inferred, and the genes in each of the two splitting branches are considered paralogous to each other (Gabaldón, 2008). The relative age of detected duplications was estimated using a phylostratigraphic approach that uses the information on which species diverged before and after the duplication node (Huerta‐Cepas and Gabaldón, 2011). Duplication frequencies at each node in the species tree were calculated by dividing the number of duplications mapped to a given node in the species tree by all the gene trees that contain that node. To calculate duplication frequencies we excluded gene trees that contained large (more than five paralogs) species‐specific expansions (expansions that contained more than five members). This filter is applied to avoid the contribution of transposon‐related gene families or pseudogenes present in the other analyzed genomes. For the rest of the analyses all duplications were considered. All orthology and paralogy relationships are available through PhylomeDB (Huerta‐Cepas *et al.*, 2014).

Gene Ontology term enrichment analysis was performed using FatiGO (Al‐Shahrour *et al.*, 2007). We compared three lists of proteins against all the other proteins encoded in the genome. The three lists were composed of the proteins involved in a duplication at the ancestral node of all *Prunus* species, the proteins specifically lost in almond and the proteins specifically lost in peach.

The trimmed alignments of 262 genes that had single‐copy orthologs in the 17 species considered were selected and concatenated. The final alignment containing 141 911 amino acid positions was used to reconstruct the maximum likelihood species tree with PhyML v.3.1 (Guindon *et al.*, 2010) using the LG amino acid substitution model and 100 bootstrap replicates. Additionally, a super‐tree was reconstructed using all trees in the phylome and a gene tree parsimony approach as implemented in duptree (Wehe *et al.*, 1889).

Divergence dates were estimated on the topology derived from the maximum likelihood approach by using the Bayesian relaxed molecular clock approach as implemented in PhyloBayes v.4.1c (Lartillot *et al.*, 2013). An uncorrelated relaxed clock model was applied, and four fossil constraints specified to the most recent common ancestor: *Prunus* (47.8 Mya; Li *et al.*, 2011), Rosaceae (98.25 Mya; Crepet and Nixon, 1996; Zhang *et al.*, 2013), the split between Fagales and Cucurbitales (84 Mya; Herendeen *et al.*, 2002; Sims *et al.*, 2002; Wikström *et al.*, 2005), Eudicots (124 Mya; Hughes and McDougall, 1990). These calibration constraints were used with soft bounds (Yang and Rannala, 2014) under a birth–death prior, and a prior on the root of the tree (183 Mya; Bell *et al.*, 2010). Two independent Markov chain Monte Carlo chains were run for 20 000 cycles, sampling posterior rates and dates every 10 cycles. The initial 25% were discarded as burn‐in. Posterior estimates of divergence dates and associated 95% credibility intervals were then computed from the remaining samples of each chain.

### Re‐sequencing of almond cultivars and comparison of almond–peach structural genome variability

Genetic variability analysis was performed on 10 traditional almond cultivars and one peach cultivar, Earlygold, that was used as an outgroup. These accessions were re‐sequenced using PE Illumina sequencing as described before. Selection of the almond lines was based on their origin as representing a range of the major areas of production in Europe (France, Spain, Italy) and the USA, and morphological characteristics (shell hardness, bloom time and self‐incompatibility) (Table S8).

The PE Illumina sequencing data from the almond cultivars were trimmed (length ≥ 35 bp, mean sliding window of 4 bp, phred quality score ≥ 20) using Trimmomatic (Bolger *et al.*, 2014). and the output was quality checked using FastQC (https://www.bioinformatics.babraham.ac.uk/projects/fastqc/). Trimmed data were aligned against the almond assembly using the BWA‐MEM algorithm v.0.7.16a‐r1181 (http://bio-bwa.sourceforge.net/bwa.shtml) with default parameters. After removal of unmapped reads, PCR duplicates and reads with mapping quality < 10 we obtained the subset of ‘clean’ reads used for variant calling, which was performed with Samtools v.1.5 (Li, 2011) with default parameters, except from the following: ‐q 10 ‐Q 20. Commands for trimming, alignment, bam filtering and variant calling can be found in the github repository (https://github.com/kostasgalexiou/sample-processing.git). Variant calling format (VCF) files were filtered by applying the following criteria: global quality ≥ 30, genotype quality ≥ 30, 8 ≤ depth ≤300, biallelic sites, minor allele frequency (MAF) ≥ 0.1. Graphical representation of variant distributions was done with Circos (Krzywinski *et al.*, 2009) in non‐overlapping windows.

Large deletions between almond and peach re‐sequencing data were identified using Pindel (Ye *et al.*, 2018) using default parameters and an insert size of 300 bp for all samples. Peach‐ and almond‐specific deletions were obtained by selecting positions with at least 20 reads/cultivar supporting the event. We also removed deletions that overlapped with N‐regions (±1000 bp) in the almond genome. For detecting variants that overlap with TEs, we considered that a position overlaps with a TE if at least one of the two elements, the deletion or the TE, had at least 80% of its sequence overlapping with the other element.

*Prunus dulcis* contigs were aligned to the *P. persica* reference genome using Nucmer from the Mummer3 package (Delcher *et al.*, 2003). Assemblytics (Nattestad and Schatz, 2016) was used to filter the alignment and detect genome‐wide variants with the following cut‐offs: unique sequence length required for considering an alignment = 10 000 bp, minimum variant size = 20 bp, maximum variant size = 25 000 bp. Structural variants were intersected with TE annotations of *P. dulcis* (insertions and repeat expansions) and *P. persica* (deletions and repeat contractions). A variant was considered to be TE‐associated when at least 50% of its sequence was spanned by a TE.

## Author contributions

PA, TA, JMC and TG designed the project and supervised research. TA, FC, LF, JGG, MG, PR and AV performed the genome assembly and annotation. JMC, AB, FB, RC, JM provided the analysis of transposable elements. TG and IJ performed the phylome analysis. KA and PA performed the variability and synteny analysis. AD, HD, AFM, MJRC, MW and WH collected materials, extracted DNA and provided re‐sequencing data. JLG and BG generated the fosmid library. PP and TA provided long‐range DNA sequencing. PA, TA, JMC, JGM, TG and KA wrote the paper.

## Conflict of interest

The authors declare no conflicts of interest.

## Supporting information


**Figure S1.** Genomescope *k*‐mer coverage model fit using 21‐mers of the Texas almond genome**.**
**Figure S2.** Synteny between the almond genome and the Texas almond × Earlygold peach linkage map.
**Figure S3.** Synteny analysis of almond versus peach genomes.
**Figure S4.** Distribution of recombination along chromosomes in almond.
**Figure S5.** Distribution of size of in‐paralog groups resulting from species‐specific duplications.
**Figure S6.** Circos graphical representation of single nucleotide polymorphism and insertion/deletion distribution across the almond genome.
**Figure S7.** Percentages of non‐transposable element (TE) and TE events for the different deletions in 10 almond varieties and one peach variety.
**Figure S8.** Single nucleotide polymorphism‐based phylogenetic analysis of 10 almond and cultivars one peach (EG2) cultivar.
**Figure S9.** Insertion time distribution of individual long terminal repeat retrotransposon families of the Copia and Gypsy superfamilies or that remained unclassified.
**Figure S10** **.** Insertion time distribution of fixed and polymorphic long terminal repeat retrotransposon insertions in peach and almond.
**Figure S11** **.** Insertion time distribution of new and orthologous long terminal repeat retrotransposon insertions in peach and almond.
**Figure S12** **.** A stacked histogram based on the 27‐mer matrix of the assembly and the paired‐end Illumina libraries.
**Figure S13** **.** Protein‐coding gene annotation pipeline.

**Table S1.** Summary of sequence data used for Texas almond genome sequencing.
**Table S2.** Comparison between the *Prunus dulcis* cv. Texas genome sequence assembly and annotation statistics and that of cv. Lauranne obtained by Sánchez‐Pérez *et al. *(2019).
**Table S3**. Mapping of single nucleotide polymorphism markers from the Texas almond × Earlygold peach linkage map onto the almond assembly.
**Table S4.** List of species used in the phylome reconstruction.
**Table S5.** Estimated dates (million years ago) and 95% highest posterior density.
**Table S6.** List of the Gene Ontology terms enriched in protein families of almond and peach that duplicated at the last common ancestor of *Prunus* species.
**Table S7.** List of the Gene Ontology terms enriched in the protein families lost specifically in peach and almond.
**Table S8**. Almond cultivars selected and their main characteristics.
**Table S9**. Mapping statistics for the re‐sequenced almond cultivars.
**Table S10** **.** Variant distribution across the almond pseudomolecules.
**Table S11**. Comparison of single nucleotide polymorphism variability parameters in *Prunus* species with whole genome sequences available.
**Table S12** **.** Deletions in 10 almond cultivars and one peach cultivar compared with the almond reference sequence and deletions that contain transposable element sequences.
**Table S13**. Summary of variants detected between *Prunus dulcis* and *Prunus persica* assemblies.
**Table S14**. General statistics of transposable element annotation in *Prunus dulcis* and *Prunus persica*.
**Table S15**. Percentage of transposable element coverage at the order level in *Prunus dulcis* and *Prunus persica*.
**Table S16**. Detailed annotation of long terminal repeat retrotransposons and miniature inverted‐repeat transposable elements in *Prunus dulcis* and *Prunus persica.*
**Table S17**. List of the 97 genes potentially involved in mesocarp development.
**Table S18** **.** Methylation status on genes potentially involved in mesocarp development and presenting transposable element insertions in peach or almond.

## Data Availability

Raw Illumina reads for the four peach cultivars (Bigtop, Earlygold, Platurno and Sweetdream), the 10 almond cultivars (Aï, Belle d’Aurons, Cristomorto, Desmayo largueta, Falsa Barese, Genco, Marcona, Nonpareil, Ripon, Vivot) and one *P. webbii* are available at the European Nucleotide Archive (ENA) under the study with a primary accession PRJEB32985 and corresponding experiment IDs ERX3390856‐ERX3390868 (10 almond cultivars; there are two entries for Ai, Belle d’Aurons and Desmayo largueta due to double Illumina runs), ERX3391776‐ERX3391779 (four peach cultivars) and ERX3391780 for *P. webbii*. Raw Illumina data for the seven peach cultivars (Armking, Belbinette, Blanvio, Catherine, Flatmoon, Nectalady and Tiffany) were downloaded from SRA, corresponding to the SRA accessions ERS1801609‐ERS1801614 and ERS1801617. Raw Illumina data for almond cultivars D05‐187 and S3067 were downloaded from SRA with the corresponding accession IDs of SRX245830 and SRX245832. Sequencing reads and assembly data of *P. dulcis* cv. Texas are available via the ENA (PRJEB32994). The assembly and annotation are additionally accessible via the Genome Database for Rosaceae (https://www.rosaceae.org/analysis/295) and the CNAG‐CRG (http://denovo.cnag.cat/almond).
