# Supplementary material for: Transposons played a major role in the diversification between the closely related almond and peach genomes: results from the almond genome sequence
Source: Plant J. 2019 Oct 22;101(2):455–72. doi: 10.1111/tpj.14538 (PMC7004133; doi:10.1111/tpj.14538)
Supplement: Supplementary file 1 — Figure S1. Genomescope k‐mer coverage model fit using 21‐mers of the Texas almond genome. Figure S2. Synteny between the almond genome and the Texas almond × Earlygold peach linkage map. Figure S3. Synteny analysis of almond versus peach genomes. Figure S4. Distribution of recombination along chromosomes in almond. Figure S5. Distribution of size of in‐paralog groups resulting from species‐specific duplications. Figure S6. Circos graphical representation of single nucleotide polymorphism and insertion/deletion distribution across the almond genome. Figure S7. Percentages of non‐transposable element (TE) and TE events for the different deletions in 10 almond varieties and one peach variety. Figure S8. Single nucleotide polymorphism‐based phylogenetic analysis of 10 almond and cultivars one peach (EG2) cultivar. Figure S9. Insertion time distribution of individual long terminal repeat retrotransposon families of the Copia and Gypsy superfamilies or that remained unclassified. Figure S10 . Insertion time distribution of fixed and polymorphic long terminal repeat retrotransposon insertions in peach and almond. Figure S11 . Insertion time distribution of new and orthologous long terminal repeat retrotransposon insertions in peach and almond. Figure S12 . A stacked histogram based on the 27‐mer matrix of the assembly and the paired‐end Illumina libraries. Figure S13 . Protein‐coding gene annotation pipeline. [file TPJ-101-455-s001.pdf]

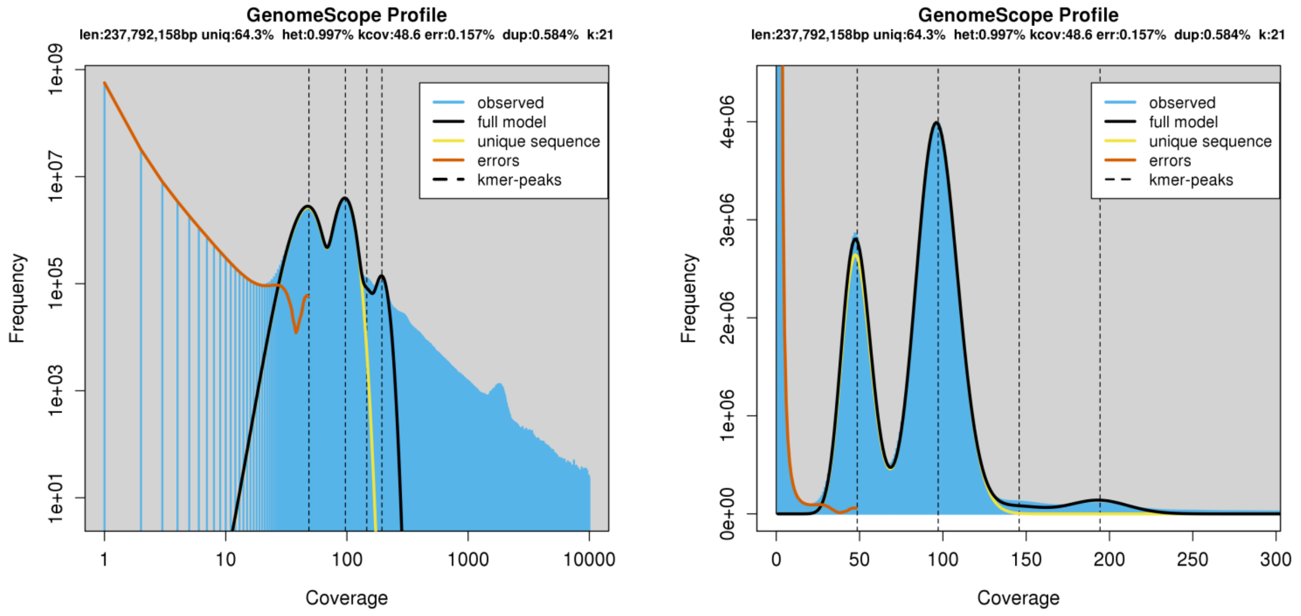

A. **B.**

Figure S1. Genomescope k-mer coverage model fit using 21-mers of the Texas almond genome. The 317 bp-insert 2x100 Illumina PE library was used for estimation. A) Both axes are shown in log-scale. The main homozygous peak is found at coverage 99, while the heterozygous peak is located at coverage 48.6. Two- and three-copy repeats are visible as well as a higher coverage peak corresponding to relatively few distinct k-mers that likely represents the chloroplast genome. B) A linear-scale plot focusing on the main and heterozygous peaks.

Figure S2. Synteny between the almond genome and the TxE linkage map.

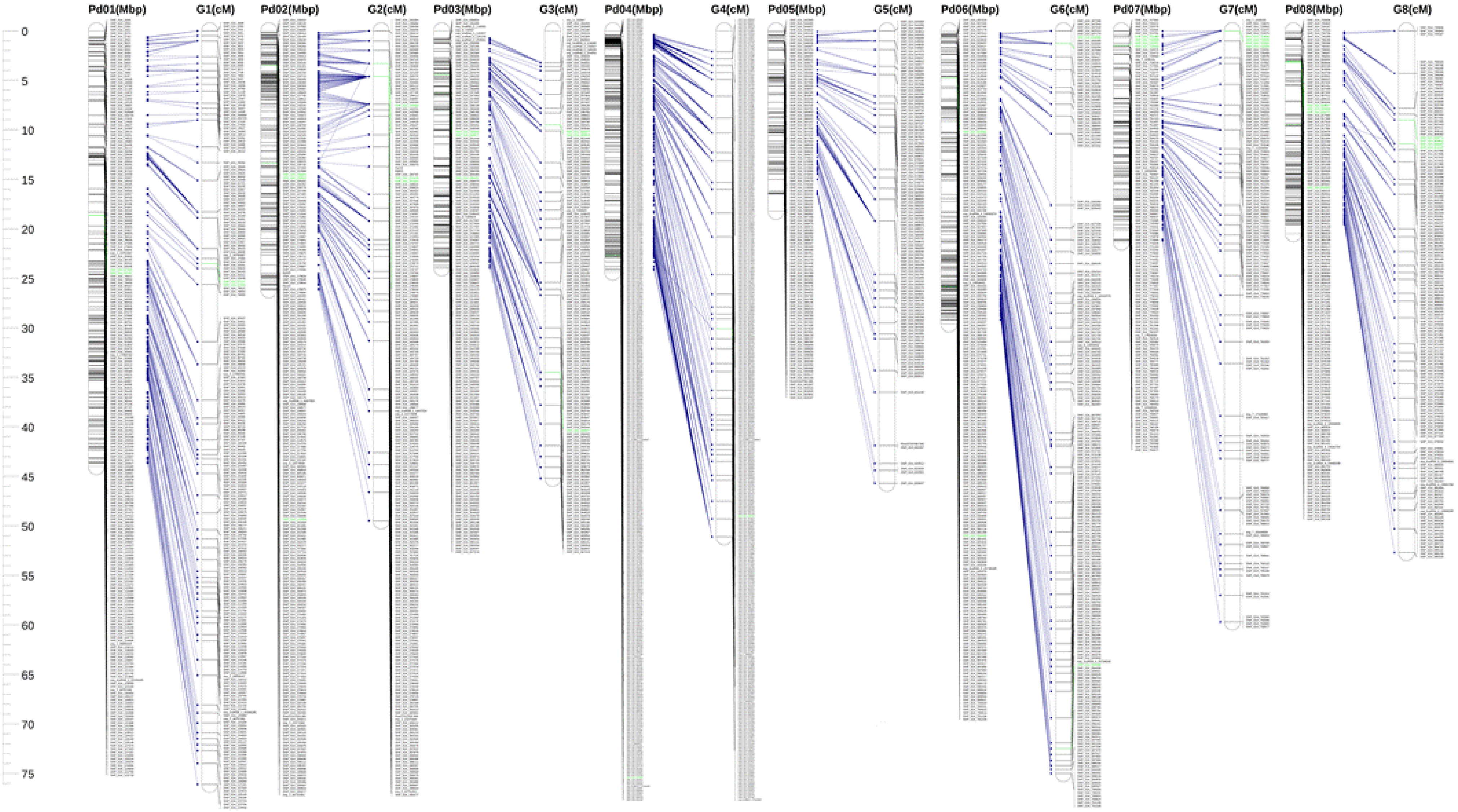

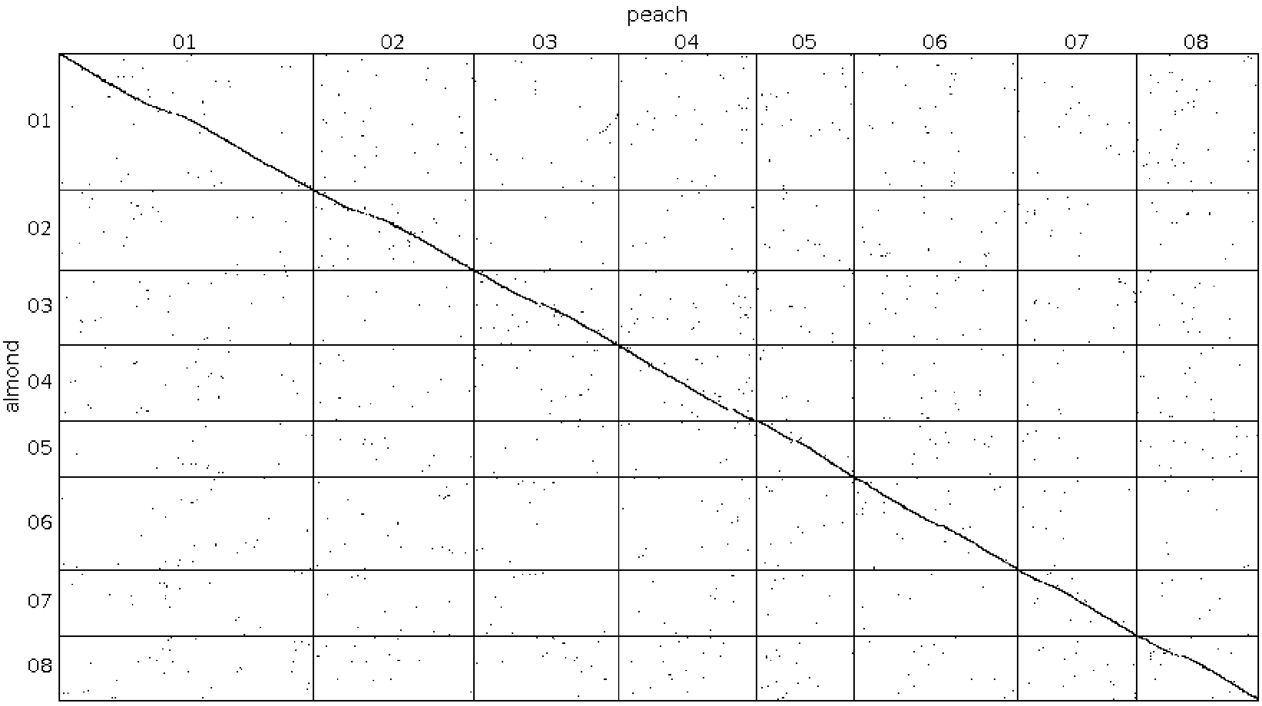

Figure S3. Synteny analysis of almond versus peach genomes.

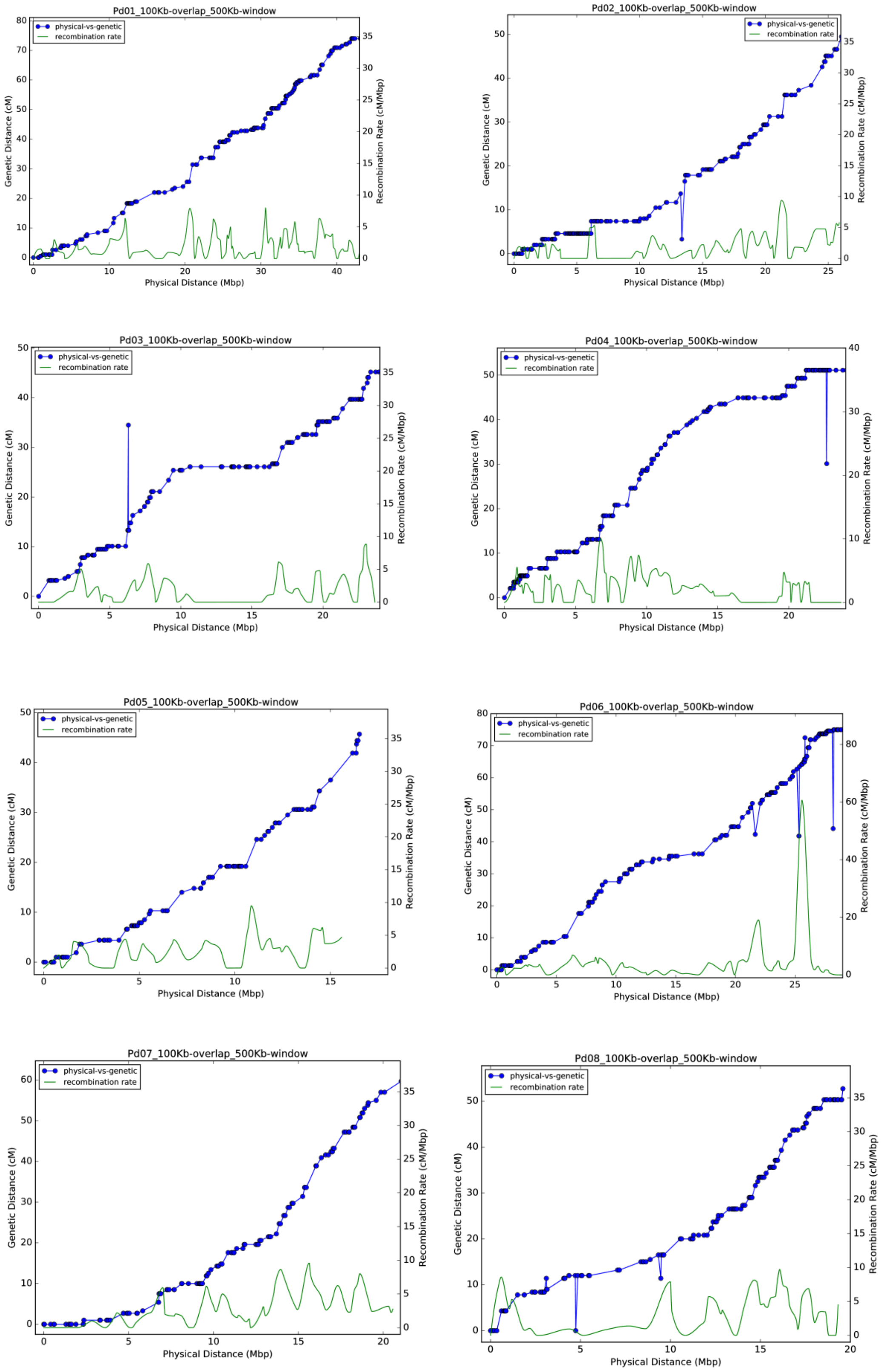

Figure S4. Distribution of recombination along chromosomes in almond. Recombination rates were calculated for 100 kb – overlapping windows with a size of 500 kb.

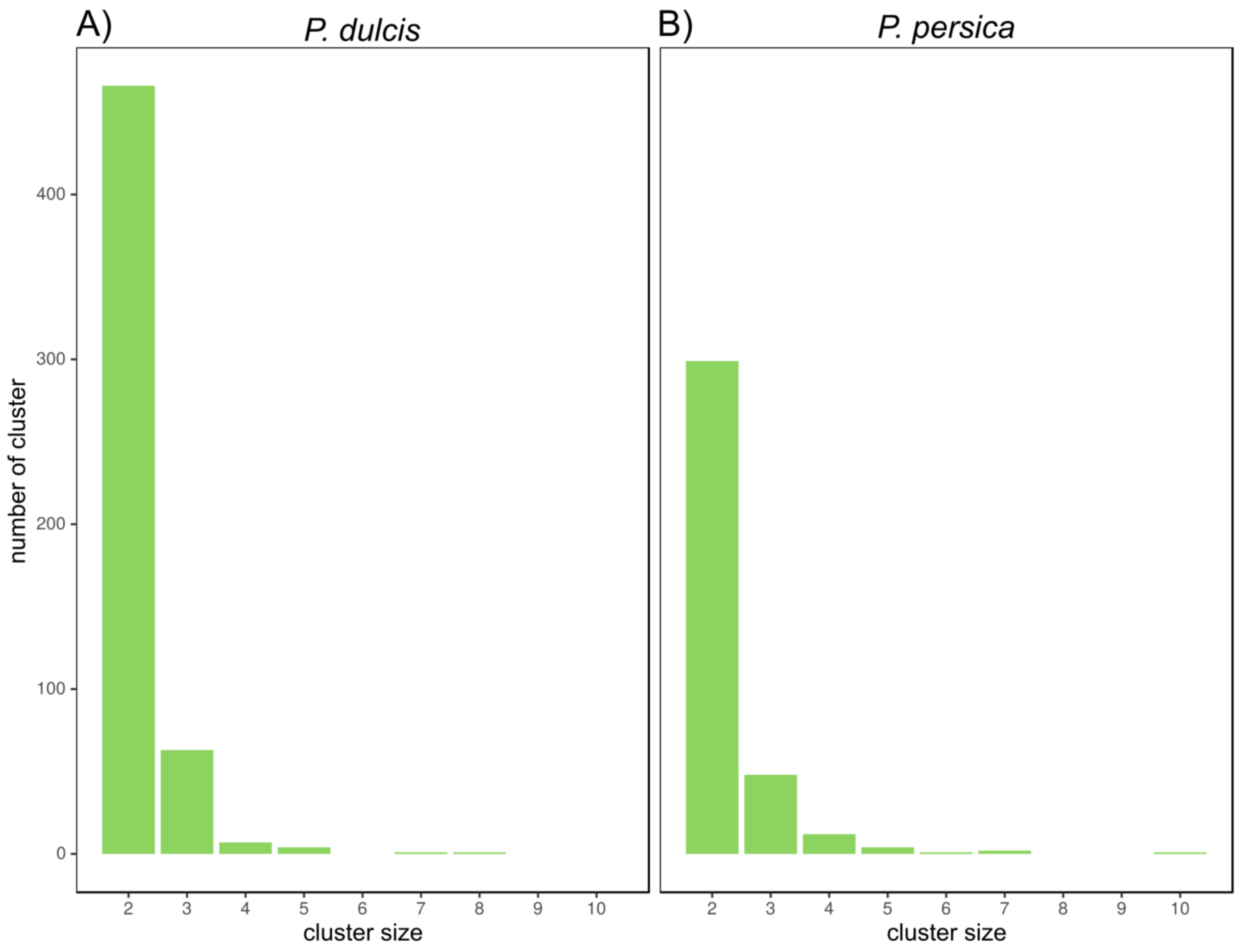

Figure S5. Distribution of size of in-paralog groups resulting from species-specific duplications. A) Almond-specific expansions. B) Peach-specific expansions.

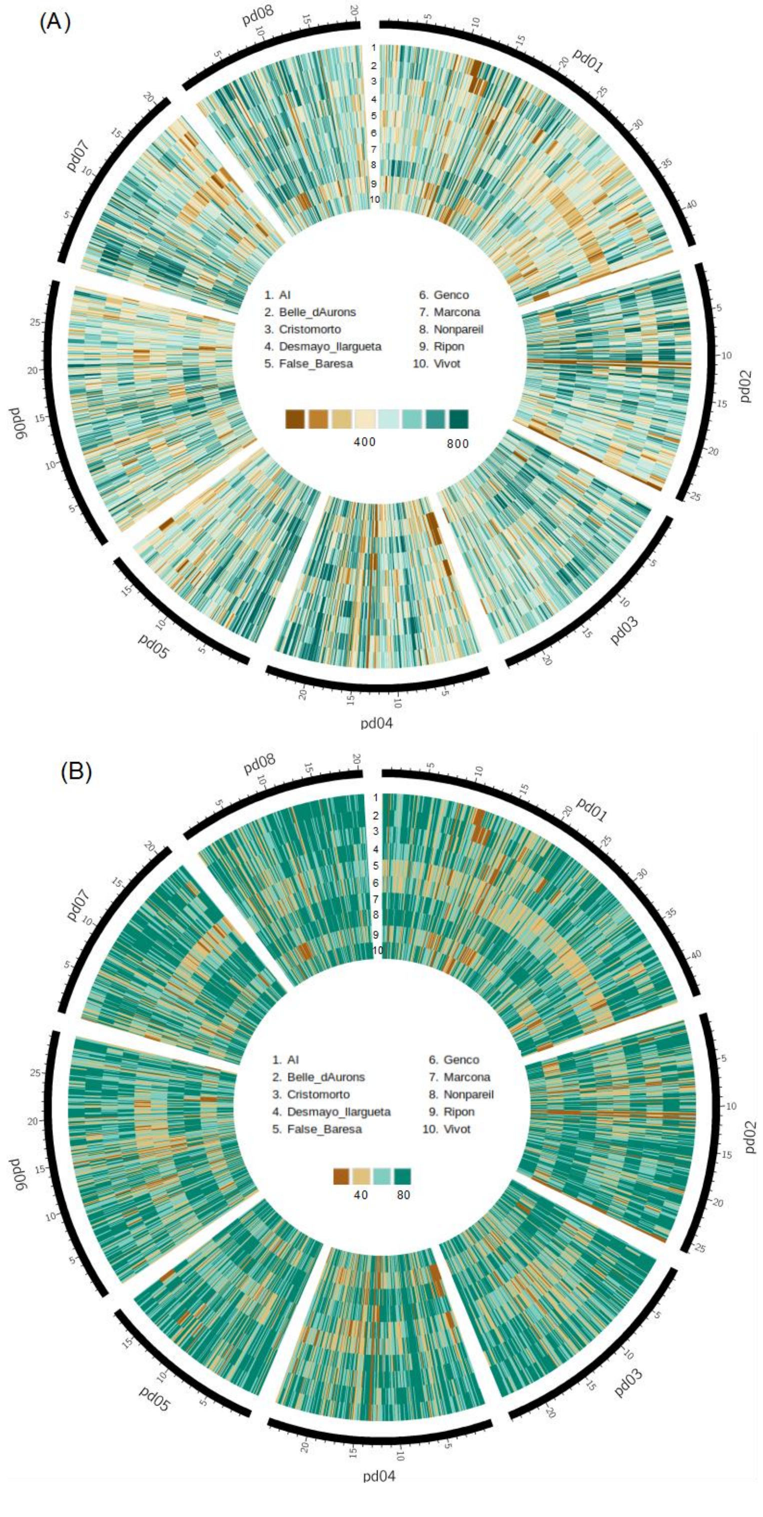

Figure S6. Circos graphical representation of SNP and INDEL distribution across the almond genome. SNPs (A) and INDELs (B) from the ten resequenced varieties were binned in windows of 100Kb and their number per window was graphically plotted.

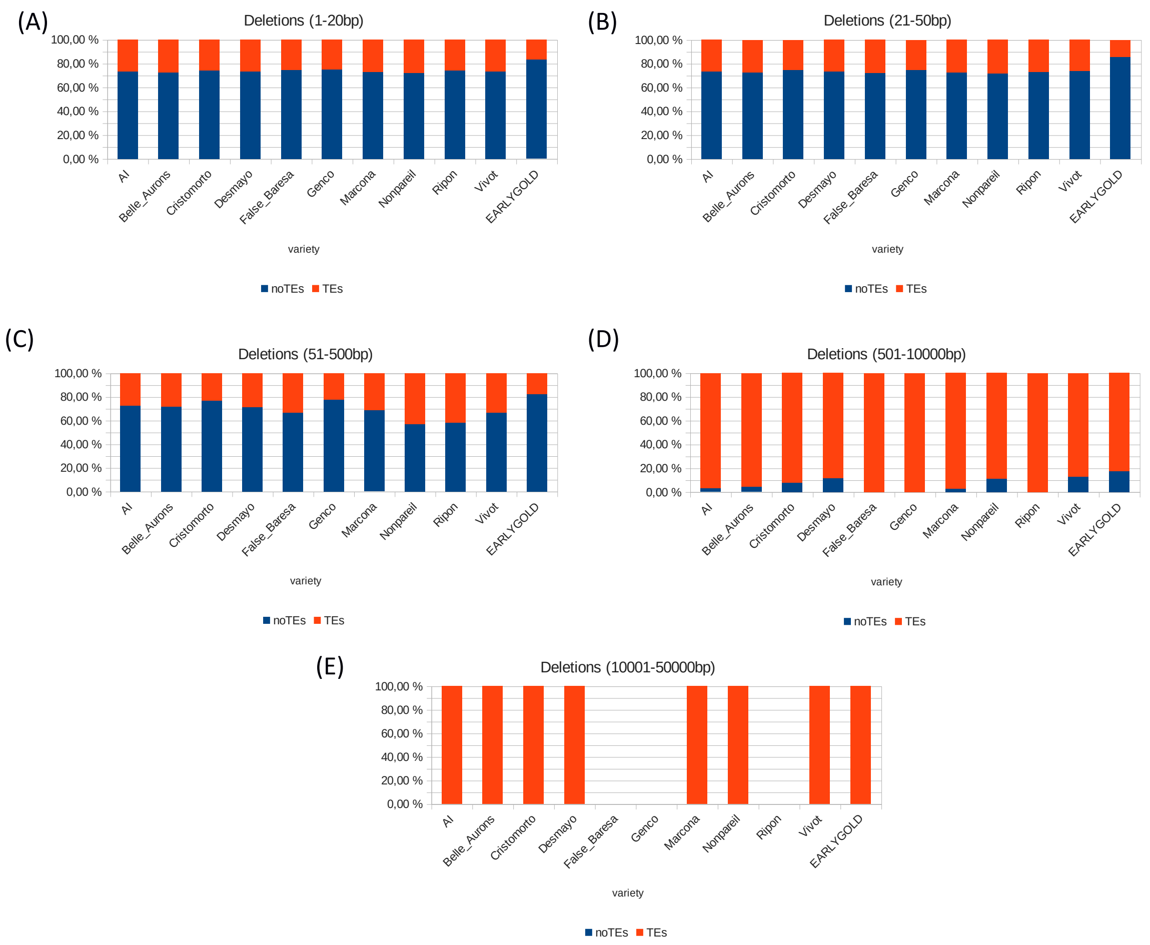

Figure S7. Percentages of non-TE and TE events for the different deletions in 10 almond varieties and 1 peach variety. (A) 1-20bp, (B) 21-50bp, (C) 51-500bp, (D) 501-10000bp and (E) 10001-50000bp.

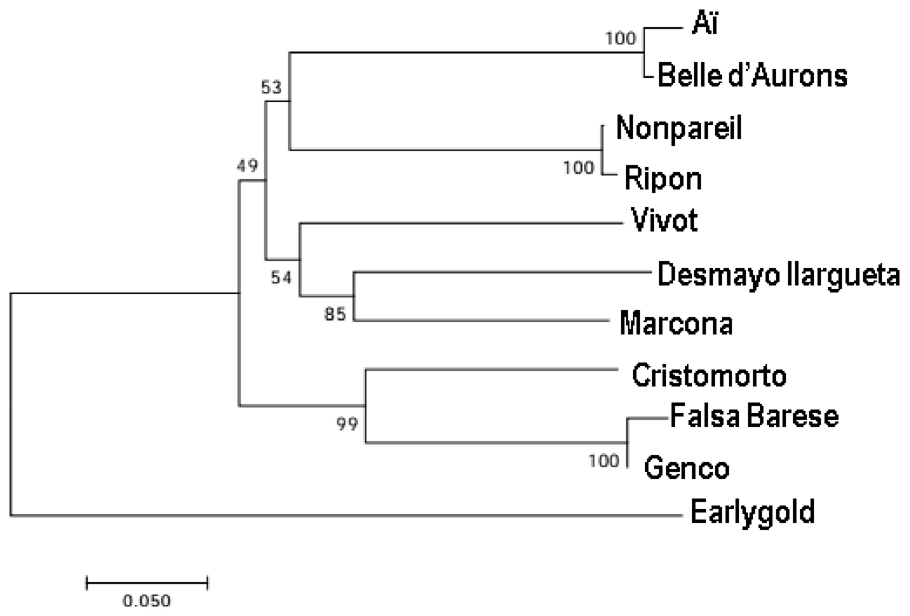

Figure S8. SNP-based phylogenetic analysis of 10 almond and one peach (Earlygold) cultivars.

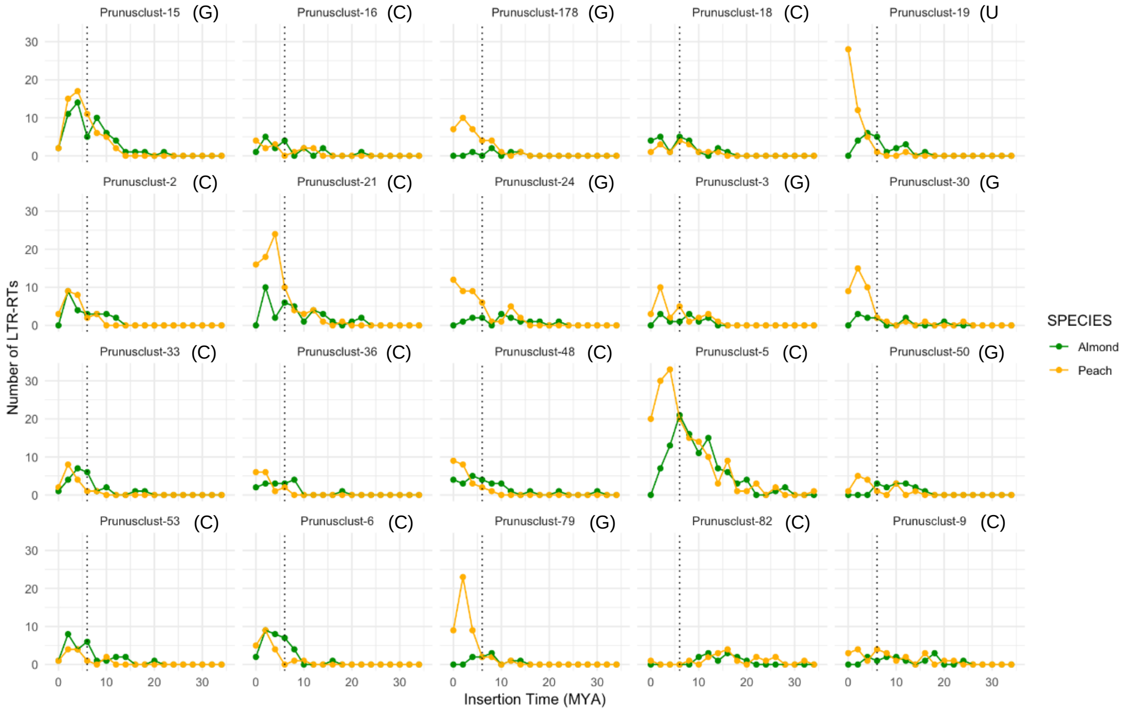

Figure S9. Insertion time distribution of individual LTR-retrotransposon families of the Copia (C) and Gypsy (G) superfamilies or that remained unclassified (U).

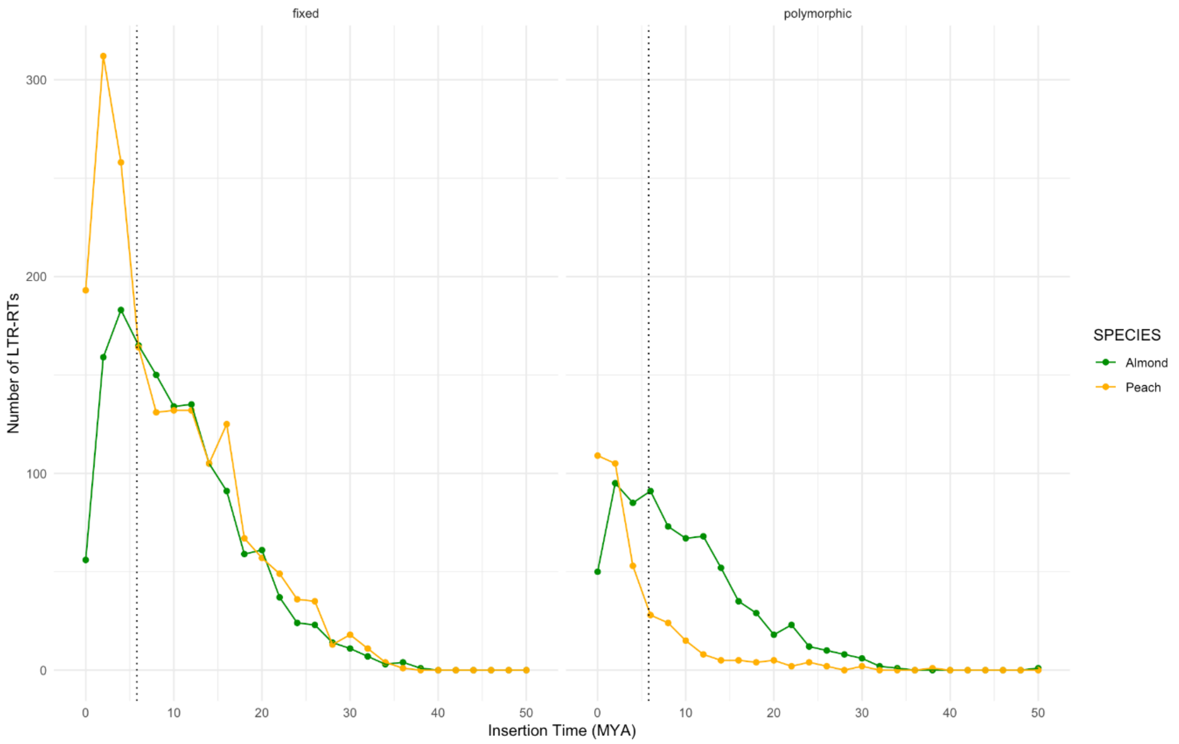

Figure S10. Insertion time distribution of fixed (left) and polymorphic (right) LTR-retrotransposon insertions in peach and almond

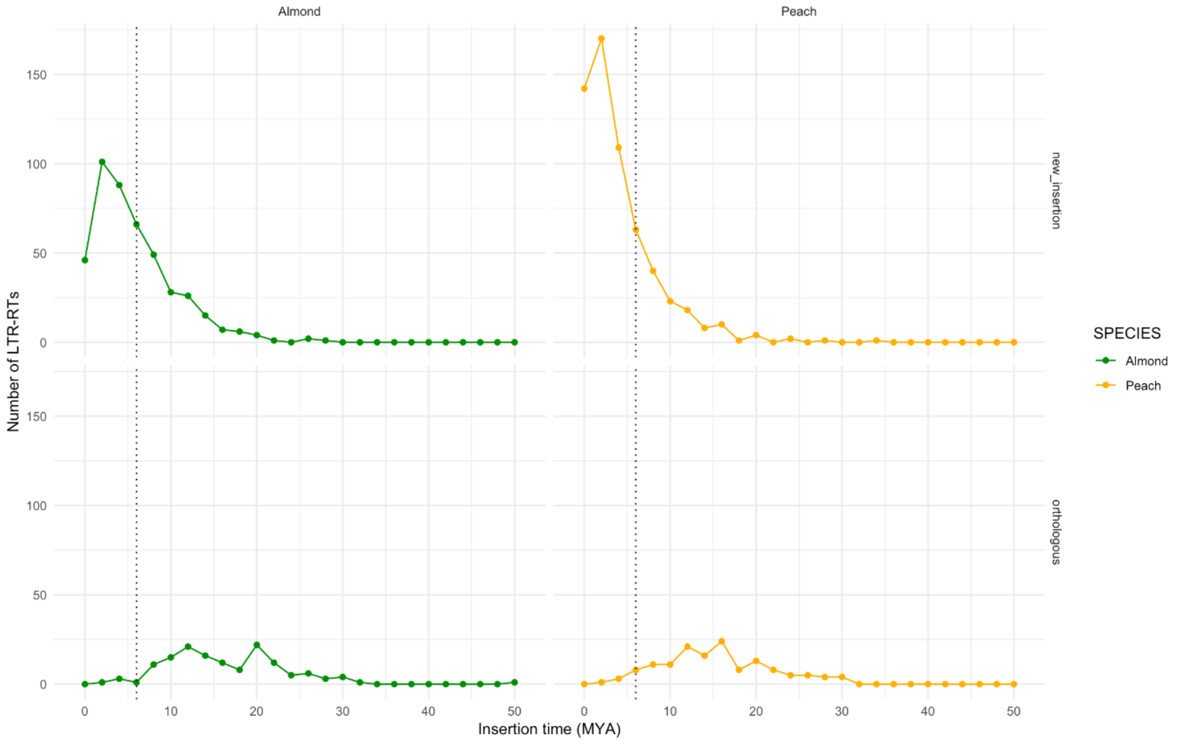

Figure S11. Insertion time distribution of new (upper panels) and orthologous (bottom panels) LTR-retrotransposon insertions in peach (left) and almond (right).

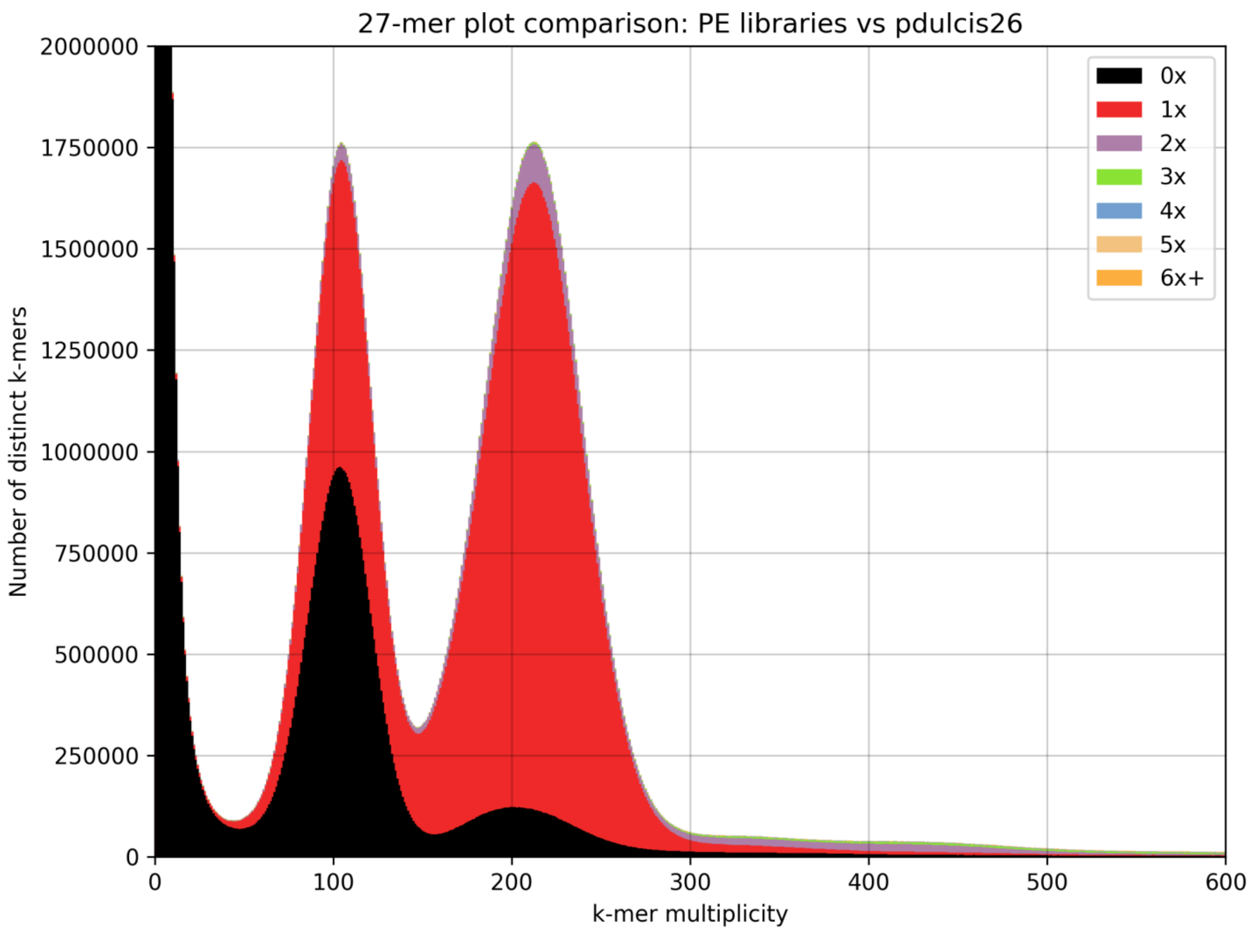

Figure S12. A stacked histogram based on the 27-mer matrix of the assembly and the paired-end Illumina libraries. The y-axis showing the number of distinct 27-mers in *Prunus dulcis* 2.0 (pdulcis26) and the x-axis the sequencing depth of each 27-mer in the PE libraries. The plot shows the amount of distinct K-mers absent from the assembly (0x class, in black), present in one copy (red), two copies (purple), and so on. As expected from a well-assembled diploid genome, half of the k-mers in the heterozygous peak (~105x) have been collapsed (the assembly contain just one allele) and the vast majority of the homozygous k-mers are present, with some missing (black) and some duplicated (purple).

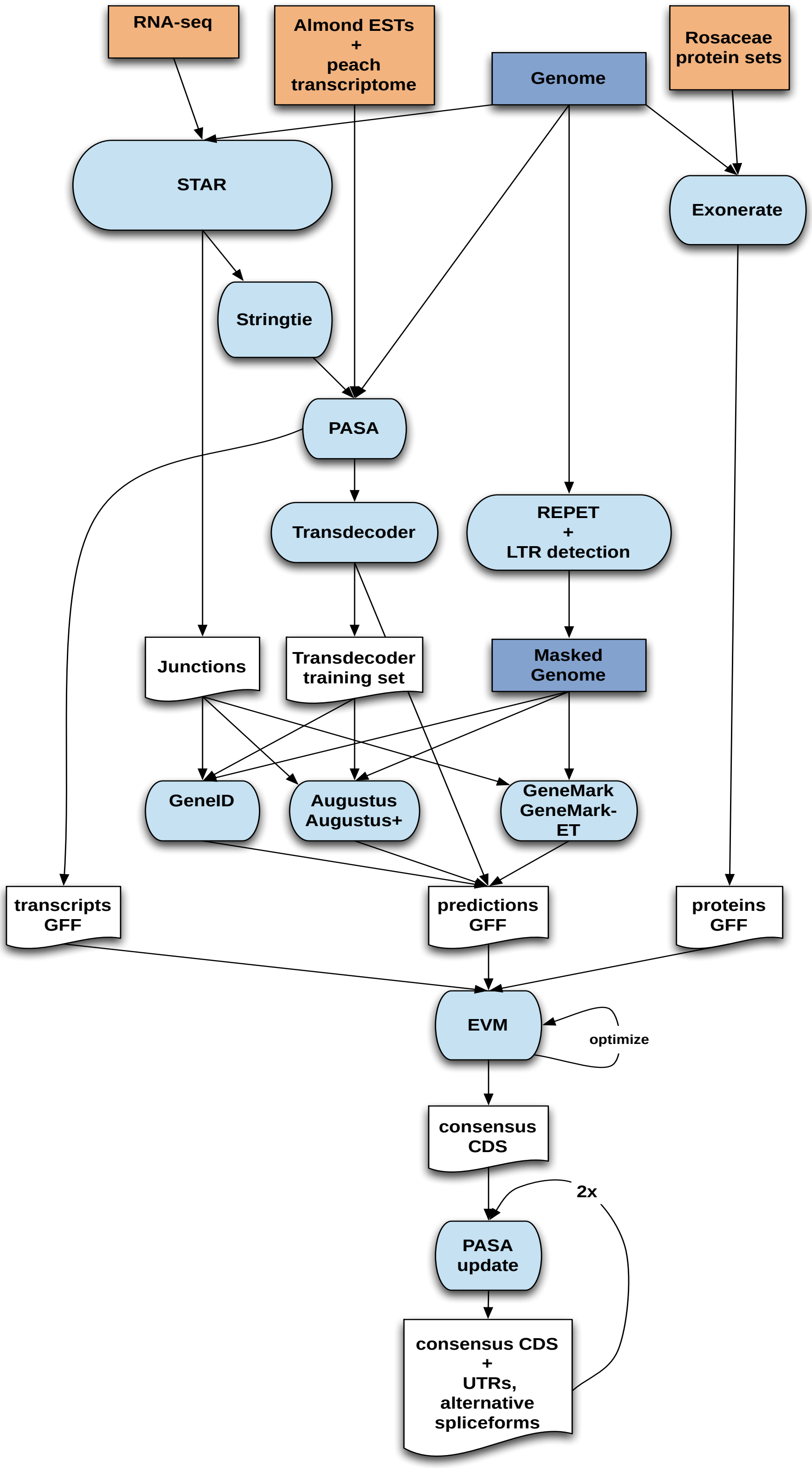

Figure S13. Protein-coding gene annotation pipeline.
