# Supplementary material for: Transposons played a major role in the diversification between the closely related almond and peach genomes: results from the almond genome sequence
Source: Plant J. 2019 Oct 22;101(2):455–72. doi: 10.1111/tpj.14538 (PMC7004133; doi:10.1111/tpj.14538)
Supplement: Supplementary file 2 — Table S1. Summary of sequence data used for Texas almond genome sequencing. Table S2. Comparison between the Prunus dulcis cv. Texas genome sequence assembly and annotation statistics and that of cv. Lauranne obtained by Sánchez‐Pérez et al. (2019). Table S3. Mapping of single nucleotide polymorphism markers from the Texas almond × Earlygold peach linkage map onto the almond assembly. Table S4. List of species used in the phylome reconstruction. Table S5. Estimated dates (million years ago) and 95% highest posterior density. Table S6. List of the Gene Ontology terms enriched in protein families of almond and peach that duplicated at the last common ancestor of Prunus species. Table S7. List of the Gene Ontology terms enriched in the protein families lost specifically in peach and almond. Table S8. Almond cultivars selected and their main characteristics. Table S9. Mapping statistics for the re‐sequenced almond cultivars. Table S10 . Variant distribution across the almond pseudomolecules. Table S11. Comparison of single nucleotide polymorphism variability parameters in Prunus species with whole genome sequences available. Table S12 . Deletions in 10 almond cultivars and one peach cultivar compared with the almond reference sequence and deletions that contain transposable element sequences. Table S13. Summary of variants detected between Prunus dulcis and Prunus persica assemblies. Table S14. General statistics of transposable element annotation in Prunus dulcis and Prunus persica. Table S15. Percentage of transposable element coverage at the order level in Prunus dulcis and Prunus persica. Table S16. Detailed annotation of long terminal repeat retrotransposons and miniature inverted‐repeat transposable elements in Prunus dulcis and Prunus persica. Table S17. List of the 97 genes potentially involved in mesocarp development. Table S18 . Methylation status on genes potentially involved in mesocarp development and presenting transposable element insertions in peach or a [file TPJ-101-455-s002.docx]

**SUPPLEMENTARY TABLES**

**Table S1.** Summary of sequence data used for Texas almond genome sequencing

| type | PE | PE | PE | MP | MP | Fosmid pool (mean) | ONT |
| --- | --- | --- | --- | --- | --- | --- | --- |
|  |  |  |  |  |  |  |  |
| Fragment size | 263 | 317 | 354 | 3.1kb | 5.2kb | 310 | HMW |
| Read length | 2 x 101 | 2 x 101 | 2 x 101 | 2 x 101 | 2 x 101 | 2 x 151/251 | N50=7.3kb |
| Yield (Gb) | 22.4 | 45.7 | 32.7 | 16.4 | 17.0 | 5.2 | 10.2 |
| Depth | 81 | 166 | 119 | 60 | 62 | >200 | 37 |
|  |  |  |  |  |  |  |  |

Table S2. Comparison between the *Prunus dulcis* cv. Texas genome sequence assembly and annotation statistics and that of cv. Lauranne obtained by Sánchez-Pérez et al. (2019)

| **Assembly Name** | **This study** | **Sánchez-Pérez et al.**  **Science (2019)** |  |  |  |  |  |  |  |  |  |
| --- | --- | --- | --- | --- | --- | --- | --- | --- | --- | --- | --- |
| Cultivar | Texas | Lauranne |  |  |  |  |  |  |  |  |  |
| Protein-coding genes | 27,969 | 27,817 |  |  |  |  |  |  |  |  |  |
| BUSCO version | version 3.0.2 | n.d.† |  |  |  |  |  |  |  |  |  |
| BUSCO database | embryophyta_odb9 | embryophyta_odb9 |  |  |  |  |  |  |  |  |  |
| BUSCO %C† | 95.4% | 75.0% |  |  |  |  |  |  |  |  |  |
| BUSCO %C [Duplicated] | 6.0% | 3.75% |  |  |  |  |  |  |  |  |  |
| Complete BUSCOs (C) | 1374 | 1080 |  |  |  |  |  |  |  |  |  |
| Complete and single-copy BUSCOs (S) | 1287 | 1026 |  |  |  |  |  |  |  |  |  |
| Complete and duplicated BUSCOs (D) | 87 | 54 |  |  |  |  |  |  |  |  |  |
| Fragmented BUSCOs (F) | 15 | 127 |  |  |  |  |  |  |  |  |  |
| Missing BUSCOs (M) | 51 | 233 |  |  |  |  |  |  |  |  |  |
| Total BUSCO groups searched | 1440 | 1440 |  |  |  |  |  |  |  |  |  |
| %Repeats (REPEAT including TEs) | 39.2% | 32.4% |  |  |  |  |  |  |  |  |  |
| %Repeats (Genomescope v1.0 k=21) | 35.70% | n.d. |  |  |  |  |  |  |  |  |  |
| Length | 228Mb | 246Mb |  |  |  |  |  |  |  |  |  |
| Contig N50 | 103kb | 82.26kb |  |  |  |  |  |  |  |  |  |
| Scaffold N50 | 381.5kb | n.d. |  |  |  |  |  |  |  |  |  |
| Pseudomolecules N50 | 24.8Mb | 21.8Mb |  |  |  |  |  |  |  |  |  |
| Total Scaffolds | 1699 | 4078 |  |  |  |  |  |  |  |  |  |
| Total Placed Scaffolds | 1554 | 2572 |  |  |  |  |  |  |  |  |  |
| Total Unplaced Scaffolds | 145 | 1506 |  |  |  |  |  |  |  |  |  |
| † n.d. = not determined |  |  |  |  |  |  |  |  |  |  |  |

**Table S3**. Mapping of SNP markers from the TxE linkage map onto the almond assembly

| **Chromosome** | **SNPs**  **in map^a^** | **SNPs in assembly^b^** | **SNPs in pseudo-**  **molecules^c^** | **Syntenic and collinear markers^d^** | **Non-syntenic**  **and non-collinear markers^e^** | **Non-collinear markers** | **Different LG (marker-LG)** |
| --- | --- | --- | --- | --- | --- | --- | --- |
| **Pd01** | 248 | 232 | 231 | 229 | 3 | SNP_IGA_67137; SNP_IGA_67265 | SNP_IGA_96232 (Pd06) |
| **Pd02** | 290 | 237 | 237 | 234 | 3 | SNP_IGA_141858;  SNP_IGA_158810;  SNP_IGA_158824 |  |
| **Pd03** | 197 | 168 | 163 | 160 | 8 | SNP_IGA_300877;  SNP_IGA_300953;  SNP_IGA_356407 | SNP_IGA_887061 (pdulcis26_s0827); SNP_IGA_330713 (pdulcis26_s0920);  SNP_IGA_356484, SNP_IGA_356701 (pdulcis26_s0416); snp_3_21905073 (pdulcis26_s0407) |
| **Pd04** | 391 | 350 | 350 | 349 | 1 | SNP_IGA_406345 |  |
| **Pd05** | 134 | 116 | 116 | 116 | 0 |  |  |
| **Pd06** | 239 | 217 | 215 | 213 | 4 | SNP_IGA_619081;  SNP_IGA_694408 | SNP_IGA_9623 (Pp01);  snp_6_21067422 (pdulcis26_s0817) |
| **Pd07** | 154 | 133 | 132 | 128 | 5 | SNP_IGA_722889;  SNP_IGA_722899;  SNP_IGA_722921;  SNP_IGA_722928 | SNP_IGA_727690 (Pd08) |
| **Pd08** | 180 | 156 | 153 | 149 | 7 | SNP_IGA_809997;SNP_IGA_809892;SNP_IGA_809771;SNP_IGA_816287 | SNP_IGA_812523 (pdulcis26_s1221);  SNP_IGA_827612 (pdulcis26_s0616);  SNP_IGA_853053 (pdulcis26_s0722) |
| **Total** | **1833** | **1609** | **1597** | **1578** | **31** |  |  |

^a^number of TxE SNPs belonging to the corresponding peach linkage group

^b^number of TxE SNPs mapped onto the almond assembly

^c^number of TxE SNPs mapped onto the corresponding almond linkage group

^d^number of TxE SNPs that have the same order onto the corresponding almond linkage group

^e^number of TxE SNPs that are mapped on the corresponding linkage group but have a different order than the TxE (“order change”) or TxE SNPs that map onto different linkage group/scaffolds (“different linkage group/scaffold”)

**Table S4.** List of species used in the phylome reconstruction.

| TaxID | Species name | Source of protein coding sequences |
| --- | --- | --- |
| FRAVE | *Fragaria vesca subsp. vesca* | JGI |
| PRUMU | *Prunus mume* | NCBI |
| AMBTC | *Amborella trichopoda* | Uniprot |
| BETVU | *Beta vulgaris* | CRG Ultrasequencing Unit |
| 225117 | *Pyrus x bretschneideri* | NCBI |
| VITVI | *Vitis vinifera* | NCBI |
| CARPA | *Carica papaya* | NCBI |
| CITLA | *Citrullus lanatus* | Cucurbit Genomics Database |
| CUCME | *Cucumis melo* | melonomics.net |
| POPTR | *Populus trichocarpa* | EnsemblPlants - Release 15 |
| ARATH | *Arabidopsis thaliana* | Ensembl Plants - Release 17 |
| MALDO | *Malus x domestica* | JGI - PhytozomeV11 |
| PRUDU | *Prunus dulcis* | Prunus dulcis genome project |
| PRUVE | *Prunus persica* | JGI - PhytozomeV11 |
| SOYBN | *Glycine max* | Ensembl Plants - Release 17 |
| ORYSJ | *Oryza sativa subsp. japonica* | Ensembl Plants - Release 22 |
| PRUAV | *Prunus avium* | NCBI |

**Table S5.** Estimated dates (Mya) and 95% highest posterior density (HPD).

| **Node†** | **meandate** | **stderr** | **inf95** | **sup95** |
| --- | --- | --- | --- | --- |
| 1 | 5.8784 | 8.17344 | 0.737743 | 32.0312 |
| 2 | 20.8363 | 19.4593 | 2.31114 | 65.1583 |
| 3 | 62.0399 | 8.63017 | 49.329 | 83.1513 |
| 4 | 81.5 | 11.5929 | 60.975 | 108.329 |
| 5 | 23.6605 | 17.3587 | 4.12562 | 69.2511 |
| 6 | 97.5383 | 10.5984 | 81.064 | 123.354 |
| 7 | 126.924 | 14.4311 | 103.573 | 158.422 |
| 8 | 114.157 | 16.593 | 83.9286 | 149.426 |
| 9 | 25.331 | 16.5652 | 5.74043 | 68.7239 |
| 10 | 137.574 | 15.3032 | 112.234 | 171.606 |
| 11 | 116.867 | 20.3396 | 74.1546 | 156.087 |
| 12 | 81.7086 | 22.8562 | 39.3539 | 127.633 |
| 13 | 150.469 | 16.6399 | 122.542 | 186.548 |
| 14 | 156.15 | 17.7069 | 125.883 | 193.866 |
| 15 | 201.166 | 21.9517 | 162.337 | 248.811 |
| 16 | 220.6 | 22.4862 | 180.6 | 267.057 |

†Node numbers correspond to nodes in Figure 1A.

**Table S6.** List of the GO terms enriched in protein families of almond and peach that duplicated at the last common ancestor of *Prunus* species.

| ***P. dulcis*** |  |  |  |  |
| --- | --- | --- | --- | --- |
| Term category | term | term level | adj. p value | term name |
| molecular_function | GO:0004812 | 1 | 9.25E-04 | aminoacyl-tRNA ligase activity |
| molecular_function | GO:0005506 | 1 | 5.76E-20 | iron ion binding |
| molecular_function | GO:0008168 | 1 | 3.65E-04 | methyltransferase activity |
| molecular_function | GO:0008171 | 1 | 6.52E-10 | O-methyltransferase activity |
| molecular_function | GO:0016705 | 1 | 3.15E-20 | oxidoreductase activity, acting on paired donors, with incorporation or reduction of molecular oxygen |
| molecular_function | GO:0016747 | 1 | 1.45E-12 | transferase activity, transferring acyl groups other than amino-acyl groups |
| molecular_function | GO:0016758 | 1 | 1.38E-05 | transferase activity, transferring hexosyl groups |
| molecular_function | GO:0020037 | 1 | 3.03E-16 | heme binding |
| biological_process | GO:0006418 | 1 | 7.65E-04 | tRNA aminoacylation for protein translation |
| biological_process | GO:0055114 | 1 | 8.35E-16 | oxidation-reduction process |
| ***P. persica*** |  |  |  |  |
| Term category | term | term level | adj. p value | term name |
| molecular_function | GO:0004812 | 1 | 3.65E-04 | aminoacyl-tRNA ligase activity |
| molecular_function | GO:0004970 | 1 | 1.53E-06 | ionotropic glutamate receptor activity |
| molecular_function | GO:0005216 | 1 | 1.11E-08 | ion channel activity |
| molecular_function | GO:0005506 | 1 | 7.50E-38 | iron ion binding |
| molecular_function | GO:0010333 | 1 | 3.81E-08 | terpene synthase activity |
| molecular_function | GO:0016491 | 1 | 6.24E-11 | oxidoreductase activity |
| molecular_function | GO:0016705 | 1 | 1.72E-39 | oxidoreductase activity, acting on paired donors, with incorporation or reduction of molecular oxygen |
| molecular_function | GO:0016758 | 1 | 3.28E-04 | transferase activity, transferring hexosyl groups |
| molecular_function | GO:0016829 | 1 | 1.15E-07 | lyase activity |
| molecular_function | GO:0020037 | 1 | 1.62E-35 | heme binding |
| molecular_function | GO:0030145 | 1 | 1.68E-05 | manganese ion binding |
| molecular_function | GO:0033926 | 1 | 9.02E-05 | glycopeptide alpha-N-acetylgalactosaminidase activity |
| molecular_function | GO:0045735 | 1 | 5.06E-05 | nutrient reservoir activity |
| molecular_function | GO:0071949 | 1 | 1.20E-05 | FAD binding |
| biological_process | GO:0006418 | 1 | 4.30E-04 | tRNA aminoacylation for protein translation |
| biological_process | GO:0006811 | 1 | 1.78E-08 | ion transport |
| biological_process | GO:0008152 | 1 | 3.45E-06 | metabolic process |
| biological_process | GO:0009733 | 1 | 2.10E-05 | response to auxin |
| biological_process | GO:0055114 | 1 | 3.18E-31 | oxidation-reduction process |

**Table S7.** List of the GO terms enriched in the protein families lost specifically in peach and almond..

| ***P. persica* losses** |  |  |  |  |
| --- | --- | --- | --- | --- |
| Term category | term | term level | adj. p value | term name |
| molecular_function | GO:0003899 | 1 | 4.95E-05 | DNA-directed 5'-3' RNA polymerase activity |
| molecular_function | GO:0016747 | 1 | 3.01E-07 | transferase activity, transferring acyl groups other than amino-acyl groups |
| cellular_component | GO:0009507 | 1 | 4.95E-05 | chloroplast |
| biological_process | GO:0006351 | 1 | 4.72E-04 | transcription, DNA-templated |
| biological_process | GO:0015986 | 1 | 4.72E-04 | ATP synthesis coupled proton transport |
|  |  |  |  |  |
| ***P. dulcis* losses** |  |  |  |  |
| Term category | term | term level | adj. p value | term name |
| molecular_function | GO:0003824 | 1 | 9.26E-04 | catalytic activity |
| molecular_function | GO:0004867 | 1 | 3.56E-04 | serine-type endopeptidase inhibitor activity |
| molecular_function | GO:0004970 | 1 | 8.81E-09 | ionotropic glutamate receptor activity |
| molecular_function | GO:0005216 | 1 | 1.10E-04 | ion channel activity |
| molecular_function | GO:0005506 | 1 | 1.03E-25 | iron ion binding |
| molecular_function | GO:0016491 | 1 | 6.07E-04 | oxidoreductase activity |
| molecular_function | GO:0016705 | 1 | 2.50E-27 | oxidoreductase activity, acting on paired donors, with incorporation or reduction of molecular oxygen |
| molecular_function | GO:0016887 | 1 | 2.63E-04 | ATPase activity |
| molecular_function | GO:0020037 | 1 | 3.89E-27 | heme binding |
| molecular_function | GO:0045735 | 1 | 5.92E-04 | nutrient reservoir activity |
| molecular_function | GO:0071949 | 1 | 1.35E-04 | FAD binding |
| cellular_component | GO:0016020 | 1 | 1.80E-06 | membrane |
| biological_process | GO:0006811 | 1 | 1.35E-04 | ion transport |
| biological_process | GO:0006869 | 1 | 5.14E-04 | lipid transport |
| biological_process | GO:0009733 | 1 | 2.23E-13 | response to auxin |
| biological_process | GO:0055114 | 1 | 3.20E-22 | oxidation-reduction process |

**Table S8**. Almond cultivars selected and their main characteristics.

| Cultivar | Origin | Pedigree | Self-incompatibility^†^ | Shell hardness | Bloom time |
| --- | --- | --- | --- | --- | --- |
| Aï | France | unknown | SI | Semi-hard | Very early |
| Belle d’Aurons | France | unknown | SI | Semi-hard | Early |
| Cristomorto | Italy | unknown | SI | Hard | Late |
| Desmayo largueta | Spain | unknown | SI | Hard | Early |
| Falsa Barese | Italy | unknown | SC | Hard | Late |
| Genco | Italy | unknown | SC | Hard | Late |
| Marcona | Spain | unknown | SI | Hard | Mid |
| Nonpareil | U.S.A. | unknown | SI | soft | Mid |
| Ripon | U.S.A. | unknown | SI | soft | Very late |
| Texas | U.S.A. | Seedling from Languedoc | SI | Semi-hard | Late |
| Vivot | Spain | unknown | SI | Hard | Early |

^†^ SI: self-incompatible; SC: self-compatible

**Table S9**. Mapping statistics for the resequenced almond, peach and *Prunus webbii* cultivars.

| **Species** | **Variety/accession** | **Total reads** | **Clean**  **reads^†^** | **% Reads mapped** | **Average depth** | **Median depth** | **% Genome coverage (≥5 reads)** |
| --- | --- | --- | --- | --- | --- | --- | --- |
| **Almond** | **Aï** | 90,435,308 | 84,889,166 | 93.87 | 43.73 | 32 | 93 |
|  | **Belle d'Aurons** | 88,461,982 | 83,002,863 | 93.83 | 42.74 | 31 | 94 |
|  | **Cristomorto** | 73,876,698 | 68,824,743 | 93.16 | 36.04 | 26 | 92 |
|  | **Desmayo Largueta** | 91,447,444 | 85,201,669 | 93.17 | 43.90 | 32 | 94 |
|  | **Falsa Barese** | 81,923,166 | 64,775,811 | 79.07 | 27.68 | 19 | 90 |
|  | **Genco** | 86,412,808 | 69,105,067 | 79.97 | 29.67 | 21 | 91 |
|  | **Marcona** | 107,453,860 | 100,455,135 | 93.49 | 51.43 | 39 | 94 |
|  | **Nonpareil** | 118,234,470 | 111,519,612 | 94.32 | 47.02 | 36 | 95 |
|  | **Ripon** | 93,785,436 | 76,095,528 | 81.14 | 32.37 | 23 | 94 |
|  | **Vivot** | 86,988,642 | 82,044,847 | 94.32 | 42.83 | 31 | 93 |
|  | **S3067** | 40,282,928 | 36,606,570 | 90.87 | 10.41 | 6 | 58 |
|  | **D05-187** | 27,094,382 | 24,671,348 | 91.06 | 7.17 | 4 | 39 |
| ***Prunus webbii*** | **P755** | 73,633,632 | 60,159,408 | 81.70 | 33.81 | 27 | 91 |
| **Peach** | **Armking** | 82,794,984 | 74,339,777 | 89.79 | 41.88 | 37 | 96 |
|  | **Belbinette** | 104,313,434 | 95,385,556 | 91.44 | 53.71 | 48 | 96 |
|  | **Bigtop** | 86,507,520 | 78,288,136 | 90.50 | 44.19 | 39 | 96 |
|  | **Blanvio** | 93,115,746 | 82,317,708 | 88.40 | 45.83 | 40 | 96 |
|  | **Catherine** | 116,083,798 | 105,792,970 | 91.13 | 59.53 | 53 | 96 |
|  | **Earlygold** | 151,479,268 | 102,872,525 | 94.55 | 126.52 | 121 | 97 |
|  | **Flatmoon** | 75,525,944 | 68,414,903 | 90.58 | 39.21 | 35 | 95 |
|  | **Nectalady** | 50,331,648 | 45,653,817 | 90.71 | 25.88 | 23 | 96 |
|  | **Platurno** | 109,714,888 | 95,912,985 | 88.22 | 53.73 | 48 | 96 |
|  | **Sweetdream** | 91,043,638 | 82,019,996 | 90.09 | 46.08 | 40 | 97 |
|  | **Tiffany** | 83,499,394 | 75,073,654 | 89.91 | 42.11 | 38 | 97 |

^†^Reads used for variant calling after removal of unmapped reads, PCR duplicates and reads with mapping quality <10

**Table S10.** Variant distribution across the almond pseudomolecules.

| **Chromo-**  **some** | **Aï** | | **Belle d’Aurons** | | **Crstomorto** | | **Desmayo largueta** | | **Falsa Barese** | | **Genco** | | **Marcona** | | **Nonpareil** | | **Ripon** | | **Vivot** | | **Total** | |
| --- | --- | --- | --- | --- | --- | --- | --- | --- | --- | --- | --- | --- | --- | --- | --- | --- | --- | --- | --- | --- | --- | --- |
|  | **SNPs** | **INDELs** | **SNPs** | **INDELs** | **SNPs** | **INDELs** | **SNPs** | **INDELs** | **SNPs** | **INDELs** | **SNPs** | **INDELs** | **SNPs** | **INDELs** | **SNPs** | **INDELs** | **SNPs** | **INDELs** | **SNPs** | **INDELs** | **SNPs** | **INDELs** |
| **Pd01** | 195.744 | 27.391 | 192.338 | 26.158 | 178.882 | 24.267 | 189.620 | 26.269 | 153.651 | 16.244 | 177.310 | 19.320 | 200.737 | 28.342 | 217.220 | 29.385 | 159.769 | 18.616 | 181.801 | 25.188 | 419.236 | 65.221 |
| **Pd02** | 124.980 | 16.908 | 133.963 | 17.652 | 111.590 | 14.725 | 128.962 | 17.090 | 117.283 | 12.161 | 123.631 | 13.122 | 133.044 | 18.196 | 137.868 | 18.168 | 106.156 | 12.052 | 124.553 | 16.719 | 291.354 | 43.476 |
| **Pd03** | 122.012 | 16.100 | 121.631 | 15.825 | 112.409 | 14.564 | 113.649 | 14.858 | 107.302 | 11.207 | 110.401 | 11.704 | 119.119 | 16.025 | 132.760 | 17.096 | 102.613 | 11.292 | 114.244 | 14.872 | 257.739 | 38.013 |
| **Pd04** | 117.533 | 15.163 | 119.899 | 15.653 | 116.139 | 14.605 | 113.879 | 14.795 | 108.292 | 10.959 | 112.335 | 11.393 | 114.720 | 15.258 | 111.697 | 14.420 | 90.142 | 9.767 | 104.974 | 13.871 | 255.126 | 36.865 |
| **Pd05** | 86.423 | 12.270 | 84.640 | 11.718 | 84.973 | 11.324 | 87.550 | 12.256 | 85.256 | 9.348 | 79.821 | 8.869 | 87.923 | 12.464 | 99.811 | 13.938 | 75.648 | 9.144 | 88.430 | 12.185 | 194.446 | 30.704 |
| **Pd06** | 138.360 | 18.782 | 138.082 | 18.613 | 122.865 | 16.121 | 135.722 | 18.075 | 122.666 | 12.794 | 123.970 | 13.065 | 129.158 | 18.102 | 151.091 | 19.931 | 117.096 | 13.233 | 134.370 | 17.733 | 298.716 | 45.013 |
| **Pd07** | 102.611 | 14.033 | 112.403 | 14.773 | 97.897 | 12.946 | 100.450 | 13.503 | 85.230 | 8.571 | 100.484 | 10.408 | 99.824 | 14.153 | 97.094 | 13.346 | 86.007 | 9.760 | 106.521 | 14.292 | 231.097 | 34.542 |
| **Pd08** | 106.511 | 14.454 | 106.297 | 14.342 | 101.401 | 13.269 | 96.351 | 12.924 | 100.206 | 10.379 | 100.522 | 10.679 | 104.484 | 14.461 | 113.319 | 14.890 | 79.686 | 9.487 | 97.146 | 13.090 | 227.841 | 34.417 |
| **Un-anchored** | 10.683 | 927 | 12.959 | 1.107 | 8.911 | 778 | 10.610 | 885 | 9.946 | 612 | 11.272 | 709 | 10.761 | 947 | 11.899 | 968 | 10.280 | 719 | 10.472 | 885 | 28.027 | 2.544 |
| **Total** | 1.004.857 | 136.028 | 1.022.212 | 135.841 | 935.067 | 122.599 | 976.793 | 130.655 | 889.832 | 92.275 | 939.746 | 99.269 | 999.770 | 137.948 | 1.072.759 | 142.142 | 827.397 | 94.070 | 962.511 | 128.835 | 2.203.582 | 330.795 |

**Table S11**: Comparison of SNP variability parameters in *Prunus* species with whole genome sequences available.

| **Parameters** | **cherry** | **peach** | **almond** | **peach** | **almond** | **almond** |
| --- | --- | --- | --- | --- | --- | --- |
| **Reference** | **Shirasawa et al. (2017)** | **Yu et al. (2018)** | | **Velasco et al. (2016)** | | **This paper** |
| **genome assembly (Mbp)** | 272.3 | 227,411,381 | 227,411,381^†^ | - | - | 227,599,157 |
| **# samples** | 6 | 22 | 15 | 13 | 13 | 10 |
| **Average density (SNP/kbp)** | 2.5 | 2.1 | 19.1 | - | - | 6.2^‡^ |
| **Average # of heterozygous** | 463,240.5 | 274,037.9 | 1.331.604,7 | - | - | 995,912.4^§^ |
| **Average heterozygosity (%)** | 0.170 | 0.121 | 0.685 | - | - | 0.438 |
| **Average Inbreeding coefficient** | - | 0.3192 | -0.0909 | 0.197 | 0.002 | -0.0017 |

|  | ^†^Yu et al. used the peach genome as reference sequence, for both peach and almond. |
| --- | --- |
|  | ^‡^ Lower SNP density, as compared to Yu et al. (2018) is due to the use of the almond genome as a reference in this paper vs. the pach genoms in Yu’s, resulting in the inclusion of homozygous almond SNPs versus the peach sequence. Usage of the pdulcis26 assembly with our data de facto excludes these homozygous SNPs from our analysis. |
|  | ^§^This number corresponds to ~⅔ of the heterozygous SNPs found by Yu et al. (1,331,604), probably due to the smaller number of almond varieties used in our analysis and/or lower inherent heterozygosity of the selected varieties. |
|  |  |
|  |  |

**Table S12.** Deletions in 10 almond and one peach cultivars compared to the almond reference sequence and deletions that contain transposable element (TE) sequences.

|  | **1-20** | | | **21-50** | | | **51-500** | | | **501-10000** | | | **10001-50000** | | | **Large deletions (>50bp)** | | | **Total** | | |
| --- | --- | --- | --- | --- | --- | --- | --- | --- | --- | --- | --- | --- | --- | --- | --- | --- | --- | --- | --- | --- | --- |
| **Line** | **all** | **TEs** | **%** | **all** | **TEs** | **%** | **all** | **TEs** | **%** | **all** | **TEs** | **%** | **all** | **TEs** | **%** | **all** | **TEs** | **%** | **all** | **TEs** | **%** |
| **ALMOND** |  |  |  |  |  |  |  |  |  |  |  |  |  |  |  |  |  |  |  |  |  |
| Aï | 65,431 | 17,578 | 26,9 | 3,445 | 914 | 26.5 | 114 | 31 | 27.2 | 30 | 29 | 96.7 | 3 | 3 | 100.0 | 147 | 63 | 42.9 | 69,023 | 18,555 | 26.9 |
| Belle d'Aurons | 65,739 | 18,096 | 27,5 | 3,387 | 924 | 27.3 | 96 | 27 | 28.1 | 22 | 21 | 95.5 | 3 | 3 | 100.0 | 121 | 51 | 42.1 | 69,247 | 19,071 | 27.5 |
| Cristomorto | 59,715 | 15,423 | 25,8 | 2,792 | 702 | 25.1 | 52 | 12 | 23.1 | 13 | 12 | 92.3 | 2 | 2 | 100.0 | 67 | 26 | 38.8 | 62,574 | 16,151 | 25.8 |
| Desmayo Largueta | 63,032 | 16,735 | 26,6 | 3,135 | 835 | 26.6 | 116 | 33 | 28.4 | 43 | 38 | 88.4 | 5 | 5 | 100.0 | 164 | 76 | 46.3 | 66,331 | 17,646 | 26.6 |
| Falsa Barese | 46,450 | 11,767 | 25,3 | 1,483 | 412 | 27.8 | 18 | 6 | 33.3 | 4 | 4 | 100.0 | - | - | - | 22 | 10 | 45.5 | 47,955 | 12,189 | 25.4 |
| Genco | 49,842 | 12,530 | 25,1 | 1,739 | 436 | 25.1 | 18 | 4 | 22.2 | 7 | 7 | 100.0 | - | - | - | 25 | 11 | 44.0 | 51,606 | 12,977 | 25.1 |
| Marcona | 66,438 | 18,136 | 27,3 | 3,378 | 924 | 27.4 | 219 | 68 | 31.1 | 74 | 72 | 97.3 | 8 | 8 | 100.0 | 301 | 148 | 49.2 | 70,117 | 19,208 | 27.4 |
| Nonpareil | 69,237 | 19,304 | 27,9 | 2,785 | 791 | 28.4 | 151 | 65 | 43.0 | 63 | 56 | 88.9 | 5 | 5 | 100.0 | 219 | 126 | 57.5 | 72,241 | 20,221 | 28.0 |
| Ripon | 46,164 | 12,016 | 26,0 | 1,799 | 487 | 27.1 | 12 | 5 | 41.7 | 5 | 5 | 100.0 | - | - | - | 17 | 10 | 58.8 | 47,980 | 12,513 | 26.1 |
| Vivot | 62,104 | 16,556 | 26,7 | 3,065 | 805 | 26.3 | 96 | 32 | 33.3 | 38 | 33 | 86.8 | 2 | 2 | 100.0 | 136 | 67 | 49.3 | 65,305 | 17,428 | 26.7 |
| **Total** | 594,152 | 158,141 | 26.6 | 27,008 | 7,230 | 26.8 | 892 | 283 | 31.7 | 299 | 277 | 92.6 | 28 | 28 | 100.0 | 1,219 | 588 | 48.2 | 622,379 | 17,429 | 26.7 |
| **Average** | 59,415 | 15,814 | 26,6 | 2,701 | 723 | 26.8 | 88 | 28 | 31.7 | 30 | 28 | 92.6 | 3 | 3 | 100.0 | 120 | 59 | 49.0 | 62,236 | 17,430 | 26.7 |
| **PEACH** |  |  |  |  |  |  |  |  |  |  |  |  |  |  |  |  |  |  |  |  |  |
| Earlygold | 120,418 | 19,778 | 16,4 | 4,283 | 609 | 14.2 | 1,090 | 190 | 17.4 | 332 | 274 | 82.5 | 14 | 14 | 100,0 | 1,436 | 478 | 33.3 | 126,137 | 12,513 | 9.9 |

**Table S13**. Summary of variants detected between *P. dulcis* and *P. persica* assemblies.

|  | **Insertion** | | | **Deletion** | | | **Repeat_expansion** | | | **Repeat_contraction** | | |
| --- | --- | --- | --- | --- | --- | --- | --- | --- | --- | --- | --- | --- |
| **Size range** | **Count** | **Total bp** | **TE related** | **Count** | **Total bp** | **TE related** | **Count** | **Total bp** | **TE related** | **Count** | **Total bp** | **TE related** |
| 20-50 bp * | 5,945 | 170,266 | N/A | 5723 | 163,874 | N/A | 79 | 2,576 | N/A | 65 | 2,276 | N/A |
| 50-500 bp | 1,644 | 223,420 | 592 | 1630 | 244,546 | 317 | 571 | 133,996 | 122 | 666 | 160,939 | 137 |
| 500-10000 bp | 472 | 947,624 | 358 | 497 | 1,204,612 | 299 | 1,099 | 2,990,754 | 266 | 1550 | 5,096,489 | 293 |
| 10000-50000 bp | 9 | 104,033 | 4 | 41 | 574,692 | 25 | 52 | 676,160 | 10 | 337 | 5,227,362 | 60 |
| Total | 8,070 | 1,445,343 | 954 | 7,929 | 2,189,624 | 641 | 1,801 | 3,803,486 | 398 | 2,618 | 10,487,066 | 490 |

* This size interval does not match with the size of any transposon family

**Table S14**. General statistics of TE annotation in *P. dulcis* and *P. persica*.

|  | *P. dulcis* | *P. persica* |
| --- | --- | --- |
| **Total TE coverage (%)** | 38.21 | 37.60 |
| **Number of consensus sequences** | 3,994 | 3,922 |
| **Consensuses with full-length copies** | 2,307 (57.7 %) | 2,116 (53.9 %) |
| **Number of copies** | 73,679 | 65,472 |
| **Number of full-length copies** | 7,966 (10.8 %) | 9,143 (14.0 %) |

**Table S15**. Percentage of TE coverage at the order level in *P. dulcis* and *P. persica*.

| TE class | TE order | *P. dulcis* | *P. persica* |
| --- | --- | --- | --- |
| **Class I** | LTR | 21.28 | 19.8 |
|  | LINE | 1.74 | 1.93 |
|  | SINE | 0.27 | 0.4 |
|  | DIRS | 0.28 | 0.33 |
| **Class II** | TIR | 13 | 14.15 |
|  | Helitron | 1.27 | 0.81 |
|  | Maverick | 0.35 | 0.18 |

**Table S16**. Detailed annotation of LTR retrotransposons and MITEs in *P. dulcis* and *P. persica.*

| LTR-retrotransposon superfamily | *P. dulcis* | *P. persica* |
| --- | --- | --- |
| **Copia** | 964 | 1,040 |
| **Gypsy** | 392 | 517 |
| **Unclassified*** | 792 | 658 |
| **TOTAL LTR-retrotransposons** | 2,148 | 2,215 |
| MITES |  |  |
| **Full length** | 10,460 | 8,738 |
| **Partial MITE copies** | 56,196 | 53,711 |
| **TOTAL MITEs** | 66,656 | 62,449 |

*Elements carrying Long Terminal Repeats but lacking one or more coding domains

**Table S17.** List of the 97 genes potentially involved in mesocarp development.

| **TAIR_name_description (Best hit)** | **TAIR_accession (Best hit)** | **Chromosome** | **Gene_accession Peach (Prupe) Almond (Prudul26A)** |
| --- | --- | --- | --- |
| Symbols: WUS, PGA6, WUS1 \| Homeodomain-like superfamily protein \| chr2:7809100-7810671 REVERSE LENGTH=292 | AT2G17950.1 | Pp07 | Prupe.7G167700 |
| Homeodomain-like superfamily protein | AT2G17950.1 | Pd07 | Prudul26A011412 |
| Symbols: WOX1 \| WUSCHEL related homeobox 1 \| chr3:6161155-6163183 REVERSE LENGTH=350 | AT3G18010.1 | Pp05 | Prupe.5G232600 |
| WUSCHEL related homeobox 1 | AT3G18010.1 | Pd05 | Prudul26A005677 |
| Symbols: WOX2 \| WUSCHEL related homeobox 2 \| chr5:23933408-23934627 REVERSE LENGTH=260 | AT5G59340.1 | Pp06 | Prupe.6G080100 |
| WUSCHEL related homeobox 2 | AT5G59340.1 | Pd06 | Prudul26A022172 |
| Symbols: PRS, WOX3, PRS1 \| Homeodomain-like superfamily protein \| chr2:12262115-12263286 FORWARD LENGTH=244 | AT2G28610.1 | Pp06 | Prupe.6G088900 |
| Homeodomain-like superfamily protein | AT2G28610.1 | Pd06 | Prudul26A028785 |
| Symbols: WOX4 \| WUSCHEL related homeobox 4 \| chr1:17236903-17237953 REVERSE LENGTH=251 | AT1G46480.1 | Pp01 | Prupe.1G432100 |
| WUSCHEL related homeobox 4 | AT1G46480.1 | Pd01 | Prudul26A007500 |
| Symbols: WOX5 \| WUSCHEL related homeobox 5 \| chr3:3527606-3528263 FORWARD LENGTH=182 | AT3G11260.1 | Pp02 | Prupe.2G247100 |
| WUSCHEL related homeobox 5 | AT3G11260.1 | Pd02 | Prudul26A002773 |
| Symbols: WOX9, HB-3, STIP \| homeobox-3 \| chr2:14341639-14343597 REVERSE LENGTH=378 | AT2G33880.1 | Pp04 | Prupe.4G055300 |
| homeobox-3 | AT2G33880.1 | Pd04 | Prudul26A031911 |
| Symbols: WOX11 \| WUSCHEL related homeobox 11 \| chr3:889515-892162 REVERSE LENGTH=268 | AT3G03660.1 | Pp07 | Prupe.7G016600 |
| WUSCHEL related homeobox 11 | AT3G03660.1 | Pd07 | Prudul26A026968 |
| Symbols: HB-4, WOX13, ATWOX13 \| WUSCHEL related homeobox 13 \| chr4:16875814-16877167 REVERSE LENGTH=268 | AT4G35550.1 | Pp05 | Prupe.5G009600 |
| WUSCHEL related homeobox 13 | AT4G35550.1 | Pd05 | Prudul26A022613 |
| Symbols: HB-4, WOX13, ATWOX13 \| WUSCHEL related homeobox 13 \| chr4:16875814-16877167 REVERSE LENGTH=268 | AT4G35550.1 | Pp07 | Prupe.7G183300 |
| WUSCHEL related homeobox 13 | AT4G35550.1 | Pd07 | Prudul26A005622 |
| Symbols: STM, BUM1, SHL, WAM1, BUM, WAM \| KNOX/ELK homeobox transcription factor \| chr1:23058796-23061722 REVERSE LENGTH=382 | AT1G62360.1 | Pp03 | Prupe.3G212100 |
| KNOX/ELK homeobox transcription factor | AT1G62360.1 | Pd03 | Prudul26A011470 |
| Symbols: STM, BUM1, SHL, WAM1, BUM, WAM \| KNOX/ELK homeobox transcription factor \| chr1:23058796-23061722 REVERSE LENGTH=382 | AT1G62360.1 | Pp04 | Prupe.4G015200 |
| KNOX/ELK homeobox transcription factor | AT1G62360.1 | Pd04 | Prudul26A016643 |
| Symbols: KNAT1, BP, BP1 \| KNOTTED-like from Arabidopsis thaliana \| chr4:5147969-5150610 REVERSE LENGTH=398 | AT4G08150.1 | Pp01 | Prupe.1G416700 |
| KNOTTED-like from Arabidopsis thaliana | AT4G08150.1 | Pd01 | Prudul26A000871 |
| Symbols: KNAT3 \| KNOTTED1-like homeobox gene 3 \| chr5:8736208-8738115 FORWARD LENGTH=431 | AT5G25220.1 | Pp01 | Prupe.1G486200 |
| KNOTTED1-like homeobox gene 3 | AT5G25220.1 | Pd01 | Prudul26A030151 |
| Symbols: KNAT3 \| KNOTTED1-like homeobox gene 3 \| chr5:8736208-8738115 FORWARD LENGTH=431 | AT5G25220.1 | Pp07 | Prupe.7G254700 |
| KNOTTED1-like homeobox gene 3 | AT5G25220.2 | Pd07 | Prudul26A023314 |
| Symbols: KNAT6, KNAT6L, KNAT6S \| KNOTTED1-like homeobox gene 6 \| chr1:8297499-8302492 REVERSE LENGTH=327 | AT1G23380.1 | Pp01 | Prupe.1G249600 |
| KNOTTED1-like homeobox gene 6 | AT1G23380.1 | Pd01 | Prudul26A030330 |
| Symbols: KNAT6, KNAT6L, KNAT6S \| KNOTTED1-like homeobox gene 6 \| chr1:8297499-8302492 REVERSE LENGTH=327 | AT1G23380.1 | Pp05 | Prupe.5G171100 |
| KNOTTED1-like homeobox gene 6 | AT1G23380.1 | Pd05 | Prudul26A026282 |
| Symbols: KNAT6, KNAT6L, KNAT6S \| KNOTTED1-like homeobox gene 6 \| chr1:8297499-8302492 REVERSE LENGTH=327 | AT1G23380.1 | Pp06 | Prupe.6G312900 |
| KNOTTED1-like homeobox gene 6 | AT1G23380.1 | Pd06 | Prudul26A020729 |
| Symbols: BEL1 \| POX (plant homeobox) family protein \| chr5:16580424-16583770 FORWARD LENGTH=611 | AT5G41410.1 | Pp05 | Prupe.5G104600 |
| POX (plant homeobox) family protein | AT5G41410.1 | Pd05 | Prudul26A029638 |
| Symbols: PHB, ATHB14, ATHB-14, PHB-1D \| Homeobox-leucine zipper family protein / lipid-binding START domain-containing protein \| chr2:14639548-14643993 REVERSE LENGTH=852 | AT2G34710.1 | Pp04 | Prupe.4G090100 |
| Homeobox-leucine zipper family protein / lipid-binding START domain-containing protein | AT2G34710.1 | Pd04 | Prudul26A027883 |
| Symbols: REV, IFL, IFL1 \| Homeobox-leucine zipper family protein / lipid-binding START domain-containing protein \| chr5:24397734-24401933 FORWARD LENGTH=842 | AT5G60690.1 | Pp06 | Prupe.6G102300 |
| Homeobox-leucine zipper family protein / lipid-binding START domain-containing protein | AT5G60690.1 | Pd06 | Prudul26A010934 |
| Symbols: ATHB-15, ATHB15, CNA, ICU4 \| Homeobox-leucine zipper family protein / lipid-binding START domain-containing protein \| chr1:19409913-19413961 REVERSE LENGTH=836 | AT1G52150.1 | Pp03 | Prupe.3G060700 |
| Homeobox-leucine zipper family protein / lipid-binding START domain-containing protein | AT1G52150.1 | Pd03 | Prudul26A003480 |
| Symbols: AG \| K-box region and MADS-box transcription factor family protein \| chr4:10383917-10388272 FORWARD LENGTH=252 | AT4G18960.1 | Pp04 | Prupe.4G070500 |
| K-box region and MADS-box transcription factor family protein | AT4G18960.1 | Pd04 | Prudul26A002883 |
| K-box region and MADS-box transcription factor family protein | AT3G58780.1 | pdulcis26_s1224 | Prudul26A010693 |
| Symbols: SHP1, AGL1 \| K-box region and MADS-box transcription factor family protein \| chr3:21739150-21741766 FORWARD LENGTH=248 | AT3G58780.1 | Pp03 | Prupe.3G170600 |
| K-box region and MADS-box transcription factor family protein | AT3G58780.1 | Pd03 | Prudul26A017925 |
| K-box region and MADS-box transcription factor family protein | AT3G58780.1 | pdulcis26_s1198 | Prudul26A028491 |
| Symbols: SEP1, AGL2 \| K-box region and MADS-box transcription factor family protein \| chr5:5151594-5153767 REVERSE LENGTH=251 | AT5G15800.1 | Pp01 | Prupe.1G290500 |
| K-box region and MADS-box transcription factor family protein | AT5G15800.1 | Pd01 | Prudul26A018154 |
| Symbols: SEP1, AGL2 \| K-box region and MADS-box transcription factor family protein \| chr5:5151594-5153767 REVERSE LENGTH=251 | AT5G15800.1 | Pp05 | Prupe.5G208400 |
| K-box region and MADS-box transcription factor family protein | AT5G15800.1 | Pd05 | Prudul26A020918 |
| Symbols: SEP2, AGL4 \| K-box region and MADS-box transcription factor family protein \| chr3:464554-466687 REVERSE LENGTH=250 | AT3G02310.1 | Pp03 | Prupe.3G249400 |
| K-box region and MADS-box transcription factor family protein | AT3G02310.1 | Pd03 | Prudul26A018991 |
| Symbols: AGL8, FUL \| AGAMOUS-like 8 \| chr5:24502736-24506013 REVERSE LENGTH=242 | AT5G60910.1 | Pp05 | Prupe.5G208500 |
| AGAMOUS-like 8 | AT5G60910.1 | Pd05 | Prudul26A029983 |
| Symbols: SEP3, AGL9 \| K-box region and MADS-box transcription factor family protein \| chr1:8593790-8595862 REVERSE LENGTH=250 | AT1G24260.1 | Pp01 | Prupe.1G223600 |
| K-box region and MADS-box transcription factor family protein | AT1G24260.1 | Pd01 | Prudul26A004046 |
| Symbols: STK, AGL11 \| K-box region and MADS-box transcription factor family protein \| chr4:6236713-6239409 REVERSE LENGTH=230 | AT4G09960.1 | Pp01 | Prupe.1G549600 |
| K-box region and MADS-box transcription factor family protein | AT4G09960.3 | Pd01 | Prudul26A020279 |
| Symbols: AP1, AGL7 \| K-box region and MADS-box transcription factor family protein \| chr1:25982576-25986102 REVERSE LENGTH=256 | AT1G69120.1 | Pp01 | Prupe.1G290600 |
| K-box region and MADS-box transcription factor family protein | AT1G69120.1 | Pd01 | Prudul26A014532 |
| Symbols: AP1, AGL7 \| K-box region and MADS-box transcription factor family protein \| chr1:25982576-25986102 REVERSE LENGTH=256 | AT1G69120.1 | Pp03 | Prupe.3G249300 |
| K-box region and MADS-box transcription factor family protein | AT1G69120.1 | Pd03 | Prudul26A004344 |
| Symbols: AP2 \| Integrase-type DNA-binding superfamily protein \| chr4:17400998-17403140 FORWARD LENGTH=432 | AT4G36920.2 | Pp06 | Prupe.6G231700 |
| Integrase-type DNA-binding superfamily protein | AT4G36920.2 | Pd06 | Prudul26A020210 |
| Symbols: AP3, ATAP3 \| K-box region and MADS-box transcription factor family protein \| chr3:20119428-20121087 REVERSE LENGTH=232 | AT3G54340.1 | Pp01 | Prupe.1G371300 |
| K-box region and MADS-box transcription factor family protein | AT3G54340.1 | Pd01 | Prudul26A001409 |
| Symbols: AP3, ATAP3 \| K-box region and MADS-box transcription factor family protein \| chr3:20119428-20121087 REVERSE LENGTH=232 | AT3G54340.1 | Pp07 | Prupe.7G164100 |
| K-box region and MADS-box transcription factor family protein | AT3G54340.1 | Pd07 | Prudul26A012600 |
| Symbols: PI \| K-box region and MADS-box transcription factor family protein \| chr5:6829203-6831208 FORWARD LENGTH=208 | AT5G20240.1 | Pp01 | Prupe.1G489400 |
| K-box region and MADS-box transcription factor family protein | AT5G20240.1 | Pd01 | Prudul26A023954 |
| Symbols: ER, QRP1 \| Leucine-rich receptor-like protein kinase family protein \| chr2:11208367-11213895 REVERSE LENGTH=976 | AT2G26330.1 | Pp01 | Prupe.1G403600 |
| Leucine-rich receptor-like protein kinase family protein | AT2G26330.1 | Pd01 | Prudul26A000395 |
| Symbols: BAM1 \| Leucine-rich receptor-like protein kinase family protein \| chr5:26281826-26284945 FORWARD LENGTH=1003 | AT5G65700.2 | Pp01 | Prupe.1G579200 |
| Leucine-rich receptor-like protein kinase family protein | AT5G65700.2 | Pd01 | Prudul26A002620 |
| Symbols: BAM1 \| Leucine-rich receptor-like protein kinase family protein \| chr5:26281826-26284945 FORWARD LENGTH=1003 | AT5G65700.2 | Pp06 | Prupe.6G212200 |
| Leucine-rich receptor-like protein kinase family protein | AT5G65700.2 | pdulcis26_s0477 | Prudul26A026055 |
| Symbols: BAM1 \| Leucine-rich receptor-like protein kinase family protein \| chr5:26281826-26284945 FORWARD LENGTH=1003 | AT5G65700.2 | Pp07 | Prupe.7G012300 |
| Leucine-rich receptor-like protein kinase family protein | AT5G65700.2 | Pd07 | Prudul26A015491 |
| Symbols: BAM3 \| Leucine-rich receptor-like protein kinase family protein \| chr4:10949822-10952924 FORWARD LENGTH=992 | AT4G20270.1 | Pp04 | Prupe.4G121500 |
| Leucine-rich receptor-like protein kinase family protein | AT4G20270.1 | Pd04 | Prudul26A014996 |
| Symbols: CLE12 \| CLAVATA3/ESR-RELATED 12 \| chr1:25841079-25841435 REVERSE LENGTH=118 | AT1G68795.1 | Pp01 | Prupe.1G304500 |
| CLAVATA3/ESR-RELATED 12 | AT1G68795.1 | Pd01 | Prudul26A026430 |
| Symbols: CLE13 \| CLAVATA3/ESR-RELATED 13 \| chr1:27815822-27816145 FORWARD LENGTH=107 | AT1G73965.1 | Pp05 | Prupe.5G216700 |
| CLAVATA3/ESR-RELATED 13 | AT1G73965.1 | Pd05 | Prudul26A002184 |
| Symbols: CLE25 \| CLAVATA3/ESR-RELATED 25 \| chr3:10670220-10670931 REVERSE LENGTH=81 | AT3G28455.1 | Pp03 | Prupe.3G275200 |
| CLAVATA3/ESR-RELATED 25 | AT3G28455.1 | Pd03 | Prudul26A017454 |
| Symbols: CLV2, AtRLP10 \| Leucine-rich repeat (LRR) family protein \| chr1:24286943-24289105 FORWARD LENGTH=720 | AT1G65380.1 | Pp06 | Prupe.6G359800 |
| Leucine-rich repeat (LRR) family protein | AT1G65380.1 | Pd06 | Prudul26A032984 |
| Symbols: CLV1, FAS3, FLO5, ATCLV1 \| Leucine-rich receptor-like protein kinase family protein \| chr1:28463631-28466652 REVERSE LENGTH=980 | AT1G75820.1 | Pp06 | Prupe.6G163000 |
| Leucine-rich receptor-like protein kinase family protein | AT1G75820.1 | Pd06 | Prudul26A005860 |
| Symbols: CLV1, FAS3, FLO5, ATCLV1 \| Leucine-rich receptor-like protein kinase family protein \| chr1:28463631-28466652 REVERSE LENGTH=980 | AT1G75820.1 | Pp01 | Prupe.1G363300 |
| Leucine-rich receptor-like protein kinase family protein | AT1G75820.1 | Pd01 | Prudul26A026004 |
| Symbols: RPK2, TOAD2, CLI1 \| receptor-like protein kinase 2 \| chr3:380726-384181 FORWARD LENGTH=1151 | AT3G02130.1 | Pp01 | Prupe.1G287800 |
| receptor-like protein kinase 2 | AT3G02130.1 | Pd01 | Prudul26A023126 |
| Symbols: RPK2, TOAD2, CLI1 \| receptor-like protein kinase 2 \| chr3:380726-384181 FORWARD LENGTH=1151 | AT3G02130.1 | Pp03 | Prupe.3G254600 |
| receptor-like protein kinase 2 | AT3G02130.1 | Pd03 | Prudul26A008064 |
| Symbols: HEC1 \| basic helix-loop-helix (bHLH) DNA-binding superfamily protein \| chr5:26766276-26767001 FORWARD LENGTH=241 | AT5G67060.1 | Pp01 | Prupe.1G434000 |
| basic helix-loop-helix (bHLH) DNA-binding superfamily protein | AT5G67060.1 | Pd01 | Prudul26A010928 |
| Symbols: HEC2 \| basic helix-loop-helix (bHLH) DNA-binding superfamily protein \| chr3:18657423-18658118 REVERSE LENGTH=231 | AT3G50330.1 | Pp07 | Prupe.7G132300 |
| basic helix-loop-helix (bHLH) DNA-binding superfamily protein | AT3G50330.1 | Pd07 | Prudul26A023559 |
| Symbols: IND1, GT140, IND, EDA33 \| basic helix-loop-helix (bHLH) DNA-binding superfamily protein \| chr4:42601-43197 REVERSE LENGTH=198 | AT4G00120.1 | Pp06 | Prupe.6G159200 |
| basic helix-loop-helix (bHLH) DNA-binding superfamily protein | AT4G00120.1 | Pd06 | Prudul26A002140 |
| Symbols: SPT \| basic helix-loop-helix (bHLH) DNA-binding superfamily protein \| chr4:17414167-17415945 FORWARD LENGTH=373 | AT4G36930.1 | Pp01 | Prupe.1G432800 |
| basic helix-loop-helix (bHLH) DNA-binding superfamily protein | AT4G36930.1 | Pd01 | Prudul26A026016 |
| Symbols: SPT \| basic helix-loop-helix (bHLH) DNA-binding superfamily protein \| chr4:17414167-17415945 FORWARD LENGTH=373 | AT4G36930.1 | Pp07 | Prupe.7G131400 |
| basic helix-loop-helix (bHLH) DNA-binding superfamily protein | AT4G36930.1 | Pd07 | Prudul26A016794 |
| Symbols: ARF1 \| auxin response factor 1 \| chr1:21980414-21984193 FORWARD LENGTH=660 | AT1G59750.4 | Pp01 | Prupe.1G585200 |
| auxin response factor 1 | AT1G59750.4 | Pd01 | Prudul26A011950 |
| Symbols: ARF1 \| auxin response factor 1 \| chr1:21980414-21984193 FORWARD LENGTH=665 | AT1G59750.3 | Pp02 | Prupe.2G190400 |
| auxin response factor 9 | AT4G23980.2 | Pd02 | Prudul26A022734 |
| Symbols: ARF1 \| auxin response factor 1 \| chr1:21980414-21984193 FORWARD LENGTH=662 | AT1G59750.2 | Pp08 | Prupe.8G252300 |
| auxin response factor 1 | AT1G59750.2 | Pd08 | Prudul26A013138 |
| Symbols: ARF2, ARF1-BP, HSS, ORE14 \| auxin response factor 2 \| chr5:24910859-24914680 FORWARD LENGTH=859 | AT5G62000.3 | Pp05 | Prupe.5G143100 |
| auxin response factor 2 | AT5G62000.3 | Pd05 | Prudul26A008717 |
| Symbols: ETT, ARF3 \| Transcriptional factor B3 family protein / auxin-responsive factor AUX/IAA-related \| chr2:14325444-14328613 REVERSE LENGTH=608 | AT2G33860.1 | Pp04 | Prupe.4G053800 |
| Transcriptional factor B3 family protein / auxin-responsive factor AUX/IAA-related | AT2G33860.1 | Pd04 | Prudul26A029863 |
| Symbols: ARF4 \| auxin response factor 4 \| chr5:24308558-24312187 REVERSE LENGTH=788 | AT5G60450.1 | Pp06 | Prupe.6G097700 |
| auxin response factor 4 | AT5G60450.1 | Pd06 | Prudul26A012274 |
| Symbols: MP, ARF5, IAA24 \| Transcriptional factor B3 family protein / auxin-responsive factor AUX/IAA-related \| chr1:6887353-6891182 FORWARD LENGTH=902 | AT1G19850.1 | Pp01 | Prupe.1G368300 |
| Transcriptional factor B3 family protein / auxin-responsive factor AUX/IAA-related | AT1G19850.1 | Pd01 | Prudul26A021243 |
| Symbols: ARF6 \| auxin response factor 6 \| chr1:10686125-10690036 REVERSE LENGTH=933 | AT1G30330.1 | Pp03 | Prupe.3G182900 |
| auxin response factor 6 | AT1G30330.1 | Pd03 | Prudul26A005587 |
| Symbols: ARF6 \| auxin response factor 6 \| chr1:10686125-10690036 REVERSE LENGTH=933 | AT1G30330.1 | Pp04 | Prupe.4G085900 |
| auxin response factor 6 | AT1G30330.1 | Pd04 | Prudul26A032416 |
| Symbols: ARF8, ATARF8 \| auxin response factor 8 \| chr5:14630151-14634106 FORWARD LENGTH=811 | AT5G37020.1 | Pp03 | Prupe.3G011800 |
| auxin response factor 8 | AT5G37020.1 | Pd03 | Prudul26A012714 |
| Symbols: ARF9 \| auxin response factor 9 \| chr4:12451592-12454737 FORWARD LENGTH=638 | AT4G23980.1 | Pp05 | Prupe.5G123400 |
| auxin response factor 9 | AT4G23980.1 | Pd05 | Prudul26A027105 |
| Symbols: ARF10 \| auxin response factor 10 \| chr2:12114331-12116665 FORWARD LENGTH=693 | AT2G28350.1 | Pp06 | Prupe.6G102800 |
| auxin response factor 10 | AT2G28350.1 | Pd06 | Prudul26A007330 |
| Symbols: ARF16 \| auxin response factor 16 \| chr4:14703369-14705564 REVERSE LENGTH=670 | AT4G30080.1 | Pp02 | Prupe.2G213000 |
| auxin response factor 16 | AT4G30080.1 | Pd02 | Prudul26A009326 |
| Symbols: ARF16 \| auxin response factor 16 \| chr4:14703369-14705564 REVERSE LENGTH=670 | AT4G30080.1 | Pp06 | Prupe.6G236200 |
| auxin response factor 16 | AT4G30080.1 | Pd06 | Prudul26A032786 |
| Symbols: ARF17 \| auxin response factor 17 \| chr1:29272405-29275193 FORWARD LENGTH=585 | AT1G77850.1 | Pp01 | Prupe.1G507000 |
| auxin response factor 17 | AT1G77850.1 | Pd01 | Prudul26A026715 |
| Symbols: ARF19, IAA22, ARF11 \| auxin response factor 19 \| chr1:6628395-6632779 REVERSE LENGTH=1086 | AT1G19220.1 | Pp01 | Prupe.1G065300 |
| auxin response factor 19 | AT1G19220.1 | Pd01 | Prudul26A005398 |
| Symbols: ARF19, IAA22, ARF11 \| auxin response factor 19 \| chr1:6628395-6632779 REVERSE LENGTH=1086 | AT1G19220.1 | Pp07 | Prupe.7G194200 |
| auxin response factor 19 | AT1G19220.1 | Pd07 | Prudul26A019041 |
| Symbols: ATOFP4, OFP4 \| ovate family protein 4 \| chr1:2124854-2125801 REVERSE LENGTH=315 | AT1G06920.1 | Pp06 | Prupe.6G042700 |
| ovate family protein 4 | AT1G06920.1 | Pd06 | Prudul26A007144 |
| Symbols: ATOFP5, OFP5 \| ovate family protein 5 \| chr4:10337449-10338498 FORWARD LENGTH=349 | AT4G18830.1 | Pp04 | Prupe.4G068000 |
| ovate family protein 5 | AT4G18830.1 | Pd04 | Prudul26A032029 |
| Symbols: ATOFP5, OFP5 \| ovate family protein 5 \| chr4:10337449-10338498 FORWARD LENGTH=349 | AT4G18830.1 | Pp04 | Prupe.4G014500 |
| ovate family protein 5 | AT4G18830.1 | Pd04 | Prudul26A023903 |
| Symbols: AGO1 \| Stabilizer of iron transporter SufD / Polynucleotidyl transferase \| chr1:17886285-17891892 REVERSE LENGTH=1048 | AT1G48410.1 | Pp05 | Prupe.5G241500 |
| Stabilizer of iron transporter SufD / Polynucleotidyl transferase | AT1G48410.1 | Pd05 | Prudul26A012572 |
| Symbols: AGO1 \| Stabilizer of iron transporter SufD / Polynucleotidyl transferase \| chr1:17886285-17891892 REVERSE LENGTH=1048 | AT1G48410.1 | Pp05 | Prupe.5G241600 |
| Stabilizer of iron transporter SufD / Polynucleotidyl transferase | AT1G48410.1 | Pd05 | Prudul26A019891 |
| Symbols: ZLL, AGO10 \| Stabilizer of iron transporter SufD / Polynucleotidyl transferase \| chr5:17611939-17616562 FORWARD LENGTH=988 | AT5G43810.2 | Pp01 | Prupe.1G022900 |
| Stabilizer of iron transporter SufD / Polynucleotidyl transferase | AT5G43810.2 | Pd01 | Prudul26A007562 |
| Symbols: CYCA2;1 \| cyclin a2;1 \| chr5:8815230-8817566 FORWARD LENGTH=437 | AT5G25380.1 | Pp01 | Prupe.1G467200 |
| cyclin a2;1 | AT5G25380.1 | Pd01 | Prudul26A014776 |
| Symbols: CYCA2;4 \| Cyclin A2;4 \| chr1:30214694-30216861 FORWARD LENGTH=461 | AT1G80370.1 | Pp03 | Prupe.3G075200 |
| Cyclin A2;4 | AT1G80370.1 | pdulcis26_s0670 | Prudul26A000826 |
| Cyclin A2;4 | AT1G80370.1 | Pd03 | Prudul26A009207 |
| Symbols: HAM3, ATHAM3, LOM3 \| GRAS family transcription factor \| chr4:57429-59105 REVERSE LENGTH=558 | AT4G00150.1 | Pp02 | Prupe.2G138400 |
| GRAS family transcription factor | AT4G00150.1 | Pd02 | Prudul26A006522 |
| Symbols: HAM3, ATHAM3, LOM3 \| GRAS family transcription factor \| chr4:57429-59105 REVERSE LENGTH=558 | AT4G00150.1 | Pp05 | Prupe.5G072300 |
| GRAS family transcription factor | AT4G00150.1 | Pd05 | Prudul26A022723 |
| Symbols: HAM4 \| GRAS family transcription factor \| chr4:17306060-17307520 FORWARD LENGTH=486 | AT4G36710.1 | Pp07 | Prupe.7G150900 |
| GRAS family transcription factor | AT4G36710.1 | Pd07 | Prudul26A023376 |
| Symbols: NIK1 \| NSP-interacting kinase 1 \| chr5:5224264-5227003 FORWARD LENGTH=638 | AT5G16000.1 | Pp01 | Prupe.1G299300 |
| NSP-interacting kinase 1 | AT5G16000.1 | Pd01 | Prudul26A024770 |
| Symbols: NIK1 \| NSP-interacting kinase 1 \| chr5:5224264-5227003 FORWARD LENGTH=638 | AT5G16000.1 | Pp03 | Prupe.3G244900 |
| NSP-interacting kinase 1 | AT5G16000.1 | Pd03 | Prudul26A020934 |
| Symbols: NIK3 \| NSP-interacting kinase 3 \| chr1:22383601-22386931 REVERSE LENGTH=632 | AT1G60800.1 | Pp01 | Prupe.1G249800 |
| NSP-interacting kinase 3 | AT1G60800.1 | Pd01 | Prudul26A020362 |
| Symbols: PIN1, ATPIN1 \| Auxin efflux carrier family protein \| chr1:27659772-27662876 FORWARD LENGTH=622 | AT1G73590.1 | Pp05 | Prupe.5G233100 |
| Auxin efflux carrier family protein | AT1G73590.1 | Pd05 | Prudul26A020908 |
| Symbols: PIN1, ATPIN1 \| Auxin efflux carrier family protein \| chr1:27659772-27662876 FORWARD LENGTH=622 | AT1G73590.1 | Pp07 | Prupe.7G031400 |
| Auxin efflux carrier family protein | AT1G73590.1 | Pd07 | Prudul26A000007 |
| Symbols: ULCS1, LC \| Transducin/WD40 repeat-like superfamily protein \| chr5:26466348-26468201 FORWARD LENGTH=331 | AT5G66240.2 | Pp07 | Prupe.7G167800 |
| Transducin/WD40 repeat-like superfamily protein | AT5G66240.2 | Pd07 | Prudul26A030066 |
| Symbols: CRC \| Plant-specific transcription factor YABBY family protein \| chr1:26007734-26008940 REVERSE LENGTH=181 | AT1G69180.1 | Pp01 | Prupe.1G290100 |
| Plant-specific transcription factor YABBY family protein | AT1G69180.1 | Pd01 | Prudul26A022993 |
| Symbols: PID, ABR \| Protein kinase superfamily protein \| chr2:14589934-14591557 REVERSE LENGTH=438 | AT2G34650.1 | Pp04 | Prupe.4G088000 |
| Protein kinase superfamily protein | AT2G34650.1 | Pd04 | Prudul26A020068 |
| Symbols: FTA, PLP, ATFTA, PFT/PGGT-IALPHA \| farnesyltransferase A \| chr3:21944209-21945781 FORWARD LENGTH=326 | AT3G59380.1 | Pp01 | Prupe.1G191500 |
| farnesyltransferase A | AT3G59380.1 | Pd01 | Prudul26A018857 |
| Symbols: ULT1, ULT \| Developmental regulator, ULTRAPETALA \| chr4:13985753-13987050 FORWARD LENGTH=237 | AT4G28190.1 | Pp03 | Prupe.3G232900 |
| Developmental regulator, ULTRAPETALA | AT4G28190.1 | Pd03 | Prudul26A025490 |
| Symbols: SOL2, CRN \| Protein kinase superfamily protein \| chr5:4252924-4254215 REVERSE LENGTH=401 | AT5G13290.2 | Pp01 | Prupe.1G216600 |
| Protein kinase superfamily protein | AT5G13290.2 | Pd01 | Prudul26A010287 |
| Symbols: KAPP, RAG1 \| kinase associated protein phosphatase \| chr5:6488450-6493182 FORWARD LENGTH=581 | AT5G19280.1 | Pp01 | Prupe.1G154400 |
| kinase associated protein phosphatase | AT5G19280.1 | Pd01 | Prudul26A032328 |
| Symbols: TSL \| Protein kinase superfamily protein \| chr5:7098213-7102970 FORWARD LENGTH=688 | AT5G20930.1 | Pp06 | Prupe.6G149600 |
| Protein kinase superfamily protein | AT5G20930.1 | Pd06 | Prudul26A019759 |
| Symbols: AXR1 \| NAD(P)-binding Rossmann-fold superfamily protein \| chr1:1498357-1501775 REVERSE LENGTH=540 | AT1G05180.1 | Pp03 | Prupe.3G217100 |
| NAD(P)-binding Rossmann-fold superfamily protein | AT1G05180.1 | Pd03 | Prudul26A004283 |
| Symbols: ATBARD1, BARD1 \| breast cancer associated RING 1 \| chr1:1036610-1040045 FORWARD LENGTH=713 | AT1G04020.2 | Pp08 | Prupe.8G218500 |
| breast cancer associated RING 1 | AT1G04020.1 | Pd08 | Prudul26A000820 |

**Table S18.** Methylation status on genes potentially involved in mesocarp development and presenting TE insertions in peach or almond.

|  |  |  | **Species** | **Chromosome** | **Gene** | | | | | **Transposable elements** | | | | | |
| --- | --- | --- | --- | --- | --- | --- | --- | --- | --- | --- | --- | --- | --- | --- | --- |
|  |  | **TAIR best hit** |  |  | **Accession** | **size (bp)** | **CG context (%)** | **CHG context (%)** | **CHH context (%)** | **Type of TE** | **size (bp)** | **CG context (%)** | **CHG context (%)** | **CHH context (%)** | **Position inside the gene /Distance to gene (bp)** |
| **TE insertion in *P.dulcis*** | **Inside the gene** | **Auxin Response Factor 8** (ARF8) | Almond | Pd03 | Prudul26A012714 | 12264 | 71,43 | 1,23 | 0,21 | MITE | 197 | 95,69 | 48,11 | 4,56 | Intron (exon8-exon9) |
|  |  |  | Peach | Pp03 | Prupe.3G011800 | 11090 | 80,81 | 1,12 | 0,06 |  |  |  |  |  |  |
|  | **Upstream region of the gene** | **WUSCHEL related homeobox 11** (WOX11) | Almond | Pd07 | Prudul26A026968 | 2225 | 11,48 | 3,2 | 0,4 | MITE | 110 | 78,67 | 2,17 | 0 | 485 |
|  |  |  | Peach | Pp07 | Prupe.7G016600 | 2622 | 14,09 | 2,91 | 0,58 |  |  |  |  |  |  |
|  |  | **Breast cancer Bssociated RING 1 /Repressor of WUSCHEL1** (BARD1/ROW1) | Almond | Pd08 | Prudul26A000820 | 4610 | 17,61 | 0,07 | 0,06 | MITE | 186 | 82,59 | 58,55 | 50,65 | 305 |
|  |  |  | Peach | Pp08 | Prupe.8G218500 | 4891 | 18,91 | 0,08 | 0,02 |  |  |  |  |  |  |
| **TE insertion in *P. persica*** | **Upstream region of the gene** | **FruitFull/AGAMOUS-like 8** (FUL/AGL8) | Peach | Pp05 | Prupe.5G208500 | 3324 | 3,43 | 0,16 | 0,13 | MITE | 595 | 93,84 | 52,5 | 16,06 | 199 |
|  |  |  | Almond | Pd05 | Prudul26A029983 | 2487 | 0 | 0 | 0,02 |  |  |  |  |  |  |
|  |  | **Tousled** (TSL) | Peach | Pp06 | Prupe.6G149600 | 9594 | 66,83 | 0,07 | 0,06 | DNA TE | 3164 | 93,91 | 65,74 | 10,83 | 267 |
|  |  |  | Almond | Pd06 | Prudul26A019759 | 9641 | 59,58 | 0,38 | 0,04 |  |  |  |  |  |  |
|  |  | **WUSCHEL related homeobox 11** (WOX11) | Peach | Pp07 | Prupe.7G016600 | 2622 | 14,09 | 2,91 | 0,58 | MITE | 175 | 96,86 | 65,38 | 29,9 | 157 |
|  |  |  | Almond | Pd07 | Prudul26A026968 | 2225 | 11,48 | 3,2 | 0,4 |  |  |  |  |  |  |
|  |  | **Auxin Response Factor 1** (ARF1) | Peach | Pp08 | Prupe.8G252300 | 6799 | 14,08 | 0,18 | 0,03 | MITE | 596 | 96,31 | 34,49 | 19,54 | 1061 |
|  |  |  | Almond | Pd08 | Prudul26A013138 | 6645 | 2,93 | 0,06 | 0,02 |  |  |  |  |  |  |
