# Supplementary material for: Transposons played a major role in the diversification between the closely related almond and peach genomes: results from the almond genome sequence
Source: Plant J. 2019 Oct 22;101(2):455–72. doi: 10.1111/tpj.14538 (PMC7004133; doi:10.1111/tpj.14538)
Supplement: Supplementary file 3 [file TPJ-101-455-s003.docx]

**Figure S1.** Genomescope k-mer coverage model fit using 21-mers of the Texas almond genome**.**

**Figure S2.** Synteny between the almond genome and the TxE linkage map.

**Figure S3.** Synteny analysis of almond versus peach genomes.

**Figure S4.** Distribution of recombination along chromosomes in almond.

**Figure S5.** Distribution of size of in-paralog groups resulting from species-specific duplications.

**Figure S6.** Circos graphical representation of SNP and indel distribution across the almond genome.

B

**Figure S7.** Percentages of non-TE and TE events for the different deletions in 10 almond varieties and 1 peach variety.

**Figure S8.** SNP-based phylogenetic analysis of 10 almond and one peach (EG2) cultivars.

**Figure S9.** Insertion time distribution of individual LTR-retrotransposon families of the Copia (C) and Gypsy (G) superfamilies or that remained unclassified (U).

**Figure S10.** Insertion time distribution of fixed (left) and polymorphic (right) LTR-retrotransposon insertions in peach and almond.

**Figure S11.** Insertion time distribution of new (upper panels) and orthologous (bottom panels) LTR-retrotransposon insertions in peach (left) and almond (right).

**Figure S12.** A stacked histogram based on the 27-mer matrix of the assembly and the paired-end Illumina libraries.

**Figure S13.** Protein-coding gene annotation pipeline.

**Table S1.** Summary of sequence data used for Texas almond genome sequencing

**Table S2.** Comparison between the *Prunus dulcis* cv. Texas genome sequence assembly and annotation statistics and that of cv. Lauranne obtained by Sánchez-Pérez et al. (2019)

**Table S3**. Mapping of SNP markers from the TxE linkage map onto the almond assembly

**Table S4.** List of species used in the phylome reconstruction.

**Table S5.** Estimated dates (Mya) and 95% highest posterior density (HPD).

**Table S6.** List of the GO terms enriched in protein families of almond and peach that duplicated at the last common ancestor of *Prunus* species.

**Table S7.** List of the GO terms enriched in the protein families lost specifically in peach and almond.

**Table S8**. Almond cultivars selected and their main characteristics.

**Table S9**. Mapping statistics for the resequenced almond cultivars.

**Table S10.** Variant distribution across the almond pseudomolecules.

**Table S11**: Comparison of SNP variability parameters in *Prunus* species with whole genome sequences available.

**Table S12.** Deletions in 10 almond and one peach cultivars compared to the almond reference sequence and deletions that contain transposable element (TE) sequences.

**Table S13**. Summary of variants detected between *P. dulcis* and *P. persica* assemblies.

**Table S14**. General statistics of TE annotation in *P. dulcis* and *P. persica*.

**Table S15**. Percentage of TE coverage at the order level in *P. dulcis* and *P. persica*.

**Table S16**. Detailed annotation of LTR retrotransposons and MITEs in *P. dulcis* and *P. persica.*

**Table S17**. List of the 97 genes potentially involved in mesocarp development.

**Table S18.** Methylation status on genes potentially involved in mesocarp development and presenting TE insertions in peach or almond.
